# Non‐uniform Photoinduced Unfolding of Supramolecular Polymers Leading to Topological Block Nanofibers

**DOI:** 10.1002/anie.202110224

**Published:** 2021-11-22

**Authors:** Keigo Tashiro, Kosuke Katayama, Kenta Tamaki, Luca Pesce, Nobutaka Shimizu, Hideaki Takagi, Rie Haruki, Martin J. Hollamby, Giovanni M. Pavan, Shiki Yagai

**Affiliations:** ^1^ Institute for Global Prominent Research (IGPR) Chiba University 1–33 Yayoi-cho Inage-ku Chiba 263–8522 Japan; ^2^ Division of Advanced Science and Engineering Graduate School of Science and Engineering Chiba University 1–33 Yayoi-cho Inage-ku Chiba 263–8522 Japan; ^3^ Department of Innovative Technologies University of Applied Sciences and Arts of Southern Switzerland Via La Santa 1 6962 Lugano-Viganello Switzerland; ^4^ Photon Factory Institute of Materials Structure Science High Energy Accelerator Research Organization Tsukuba 305–0801 Japan; ^5^ School of Physical and Geographical Sciences Keele University Keele Staffordshire ST55BG UK; ^6^ Department of Applied Science and Technology Politecnico di Torino Corso Duca degli Abruzzi 24 10129 Torino Italy; ^7^ Department of Applied Chemistry and Biotechnology Graduate School of Engineering Chiba University 1–33 Yayoi-cho Inage-ku Chiba 263–8522 Japan

**Keywords:** azobenzene, barbituric acid, co-polymer, supramolecular polymer, topological transition

## Abstract

Synthesis of one‐dimensional nanofibers with distinct topological (higher‐order structural) domains in the same main chain is one of the challenging topics in modern supramolecular polymer chemistry. Non‐uniform structural transformation of supramolecular polymer chains by external stimuli may enable preparation of such nanofibers. To demonstrate feasibility of this post‐polymerization strategy, we prepared a photoresponsive helically folded supramolecular polymers from a barbiturate monomer containing an azobenzene‐embedded rigid π‐conjugated scaffold. In contrast to previous helically folded supramolecular polymers composed of a more flexible azobenzene monomer, UV‐light induced unfolding of the newly prepared helically folded supramolecular polymers occurred nonuniformly, affording topological block copolymers consisting of folded and unfolded domains. The formation of such blocky copolymers indicates that the photoinduced unfolding of the helically folded structures initiates from relatively flexible parts such as termini or defects. Spontaneous refolding of the unfolded domains was observed after visible‐light irradiation followed by aging to restore fully folded structures.

## Graphical abstract

Helically folded supramolecular polymers with a high internal rigidity undergo non‐uniform unfolding upon UV‐irradiation, affording topological block nanofibers consisting of two distinct domains with helically folded and unfolded structures.

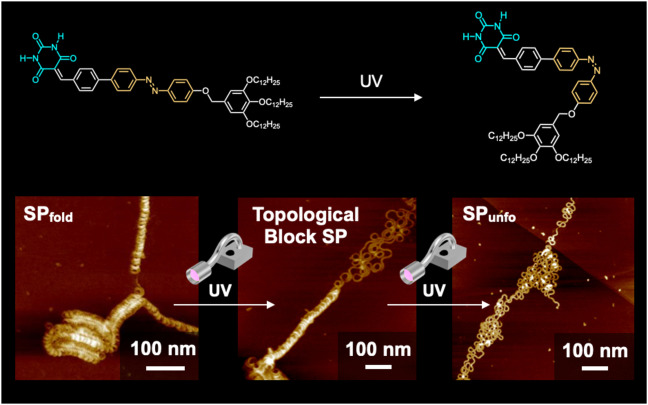

## Introduction

Modern preparation methods of polymer has enabled us to synthesize a variety of polymers with unique primary and higher order structures.^[1]^ Such well‐designed synthetic polymers are useful not only as functional soft materials wherein their collective behaviors are important, but also as more single‐chain nanomaterials that can function like biomacromolecules.^[2]^ Especially block copolymerization techniques allow the synthesis of polymers with distinct structural domains (topologies) in a main chain like proteins, which is crucial to develop polymers as discrete nanomaterials.^[3]^ For supramolecular polymers (SPs), an emerging noncovalent counterparts of polymers,^[4]^ the construction of such “topological” block or “topologically” blocky supramolecular copolymers is particularly challenging because of inherently dynamic nature in monomer binding. In other words, the monomers affording different higher‐order structures generally have different molecular structures, and accordingly it may be difficult to keep them connected in a thermodynamically stable state through non‐covalent interactions (hetero‐recognition). In fact, while several groups have recently reported elegant examples of block or blocky SPs based on thermodynamic^[5]^ or kinetic approaches,^[6]^ all of them have one‐dimensionally extended structures. As an exceptional example, we recently reported block supramolecular copolymers consisting of helically folded extended domains by kinetically controlled gradient supramolecular copolymerization of the two molecules with similar chemical structures but affording SPs with distinct higher order structures.^[7]^ However, bottom‐up design of such topological block SPs remains a formidable challenge from the viewpoint of the above compatibility between higher‐order structures and hetero‐recognition, which will limit monomer design to those leading to analogous one‐dimensional structures.

**Figure 1:**
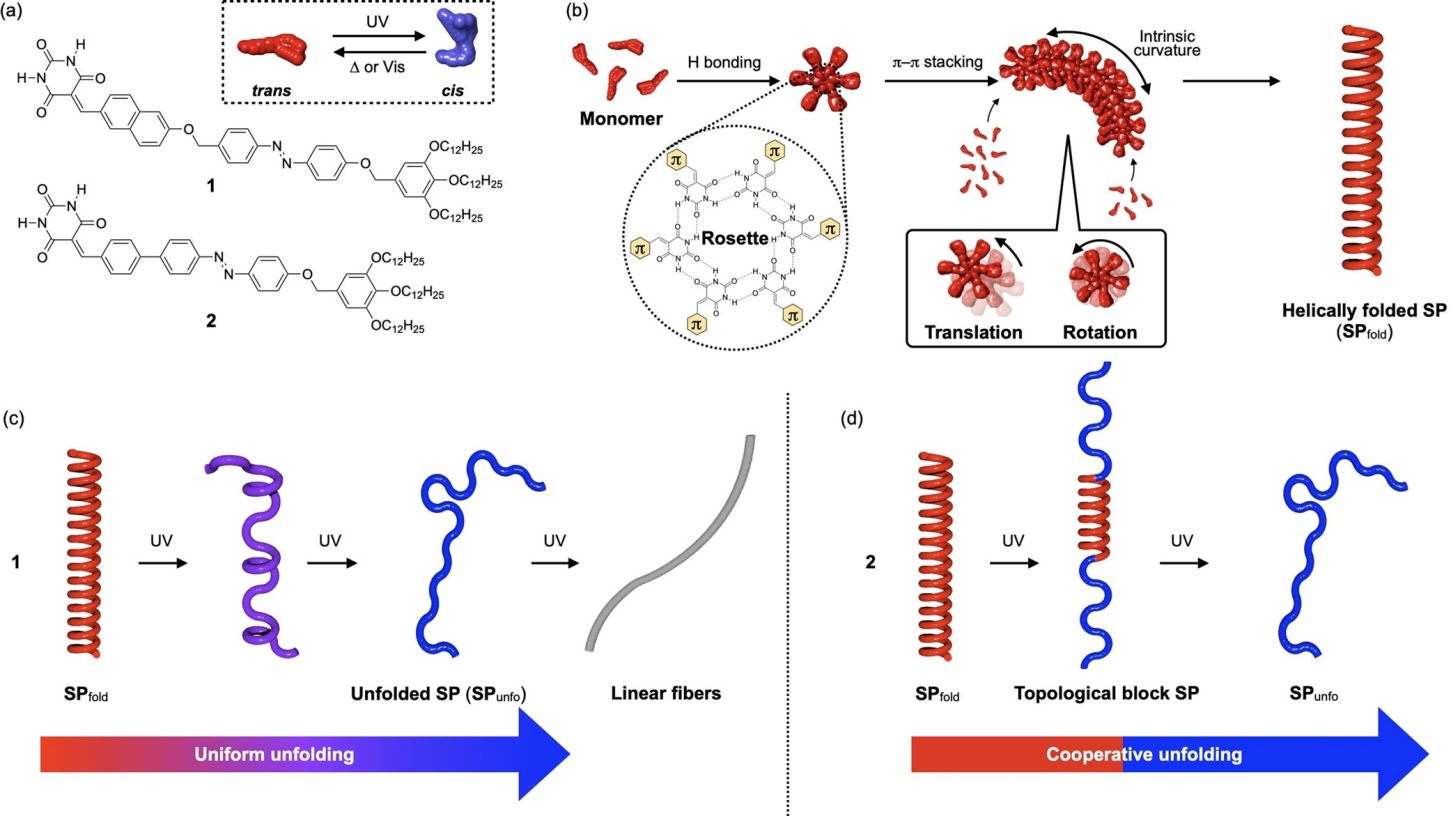
a) Molecular structures **1** and **2**. b) Formation mechanism of the **SP**_fold_. c,d) Photo‐induced unfolding processes of **SP**_fold_ composed of **1** (c) and **2** (d).

We envisage that post‐polymerization structural transformation,^[8]^ as has already been applied for covalent polymers, would be another strategy to prepare topological block SPs. As a basis that can verify this idea, we invoke the photoresponsive of monomer **1** we have reported in 2017 (Figure 1 a).^[9]^ This barbiturated azobenzene monomer forms helically folded SPs (**SP**_fold_) in nonpolar media through the formation of six‐membered hydrogen‐bonded rosettes (Figure 1 b). The helically folded structure is a result of continuous generation of intrinsic curvature upon stacking of the rosettes^[10]^ with translational and rotational displacements.^[11]^ One of the unique features of the **SP**_fold_ of **1** is unfoldability to linearly extended structures by UV‐light, which is due to the perturbation of the intrinsic curvature by the generation of sterically demanding *cis*‐azobenzene units.^[12]^ Importantly, our AFM study showed that the photoinduced unfolding proceeded uniformly throughout the entire fiber, providing unfolded SPs with curvature (**SP**_unfo_) as intermediate structures (Figure 1 c). The uniform unfolding suggests that the SP fiber of **1** is flexible enough to allow deformation of the curvature by *cis*‐azobenzene units even in tightly folded internal domains.

Based on the above mechanism, we expected that **SP**_fold_ consisting of a more rigid SP fiber allows “non‐uniform” photoinduced unfolding, and can provide blocky structures as an intermediate state (Figure 1 d). We thus designed and synthesized new monomer **2** in which azobenzene unit was embedded in a rigid π‐conjugated backbone (Figure 1 a). As we will show in this paper, **SP**_fold_ of **2** shows significant resistance to the photoinduced structural deformation at ambient temperature. At high temperature, however, photoinduced unfolding proceeds non‐uniformly, leading to topological block SPs consisting of folded and unfolded domains as intermediate structures. Our molecular dynamics simulations demonstrate that photoisomerization of azobenzene units occurred throughout entire main chains, while high inner rigidity led the cooperative unfolding.

## Results and Discussion

**Figure 2:**
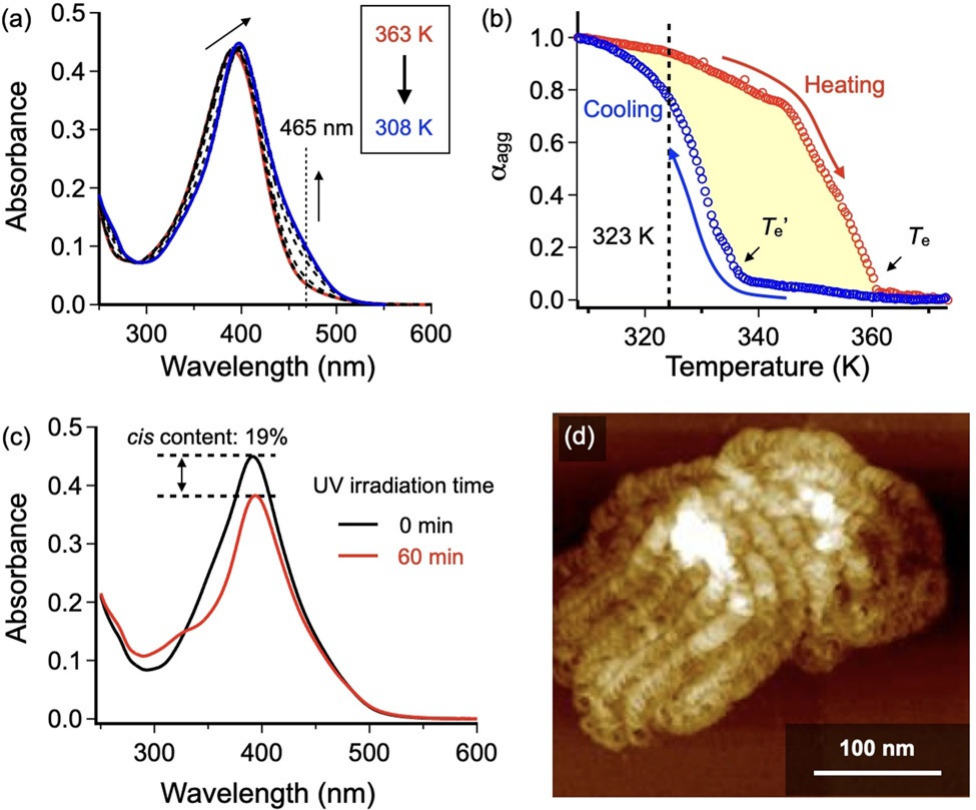
a) Temperature‐dependent UV–Vis spectra of **2** (*c=*10 μM) in MCH upon cooling from 373 to 308 K at a rate of 1.0 K min^−1^. The cooling was ceased at 308 K to avoid precipitation upon further cooling to room temperature. b) Cooling (blue) and heating (red) curves of **2** (*c=*10 μM) at a rate of 1.0 K min^−1^ obtained by plotting degree of aggregation *α*_agg_ (calculated from absorption change at 465 nm) as a function of temperature in MCH. c) UV–Vis absorption spectra of **SP**_fold_ of **2** in MCH before and after UV‐light irradiation at 308 K for 60 min. d) AFM image of the **SP**_fold_ of **2** spin‐coated onto highly oriented pyrolytic graphite (HOPG) after UV‐light irradiation in MCH at 308 K for 60 min.

Monomer **2** was synthesized according to the procedure described in the Supporting Information, and characterized by ^1^H‐ and ^13^C‐NMR spectroscopies, and APCI‐MS spectrometry. ^1^H NMR demonstrated that the azobenzene unit of as‐synthesized molecule **2** was >99.9 % *trans*‐isomer (Figure S4a). Upon cooling a hot MCH solution of monomeric **2** (*c=*10 μM) from 373 to 308 K and subsequently heating at a rate of 1.0 K min^−1^, a change of an absorption shoulder at 465 nm was reversibly observed, indicating aggregation at low temperatures (Figure 2 a). When the temperature‐dependence of this new band (*λ*=465 nm) was monitored as *α*_agg_, cooperative (nucleation‐elongation) supramolecular polymerization^[13]^ was observed for both cooling and heating curves but with different critical temperatures (*T*_e_' and *T*_e_, Figure 2 b). A gradual increase of *α*_agg_ above *T*_e′_ upon cooling is probably related to pre‐nucleation including conformational change of the π‐conjugated system such as planarization.^[14]^ The thermal hysteresis in our system is mainly caused by the formation of diverse hydrogen bonding species during cooling process, among which only discrete cyclic species (rosettes) can nucleate to form SPs (Figure S5).^[15]^ Atomic force microscopy (AFM) visualized densely folded **SP**_fold_ (Figure S6). The average radius of curvature (*r*_ave_), measured by manually fitting a circle with radius *r* along each curve, was 10.4±0.2 nm (Figure S7a).

When the above **SP**_fold_ solution was irradiated with UV‐light (*λ*=365 nm; 17 W LED lamp) at 308 K, the absorption intensity around 391 nm which is attributable to the π–π^*^ transition of the azobenzene unit decreased (Figure 2 c), indicating *trans*→*cis* photoisomerization of the azobenzene unit. Photostationary state (PSS) was achieved within 10 min, at which the percentage of *cis*‐isomer was estimated to be 19 % from a control experiment using ^1^H‐NMR (Figure S8). This ratio indicates that statistically one or two *trans*‐isomers of **2** per rosette isomerized to the *cis*‐isomers, which is similar to **1** under the same condition. For **1**, this low isomerization ratio was enough to unfold spiral structures into linearly extended fibers.^[9]^ However, no morphological change was observed for **SP**_fold_ of **2** even after prolonged UV‐light irradiation for 60 min at 308 K (Figure 2 d). Because our SPs are composed of stacked rosettes, the rigidity of monomer structures directly affect the internal rigidity (shape‐persistency) of curved SPs. Accordingly, more rigid monomer **2** should provide more rigid SPs in comparison with **1**, giving rise to a clear difference in the persistence of curvature toward the *trans*‐to‐*cis* isomerization of the azobenzene units.

**Figure 3:**
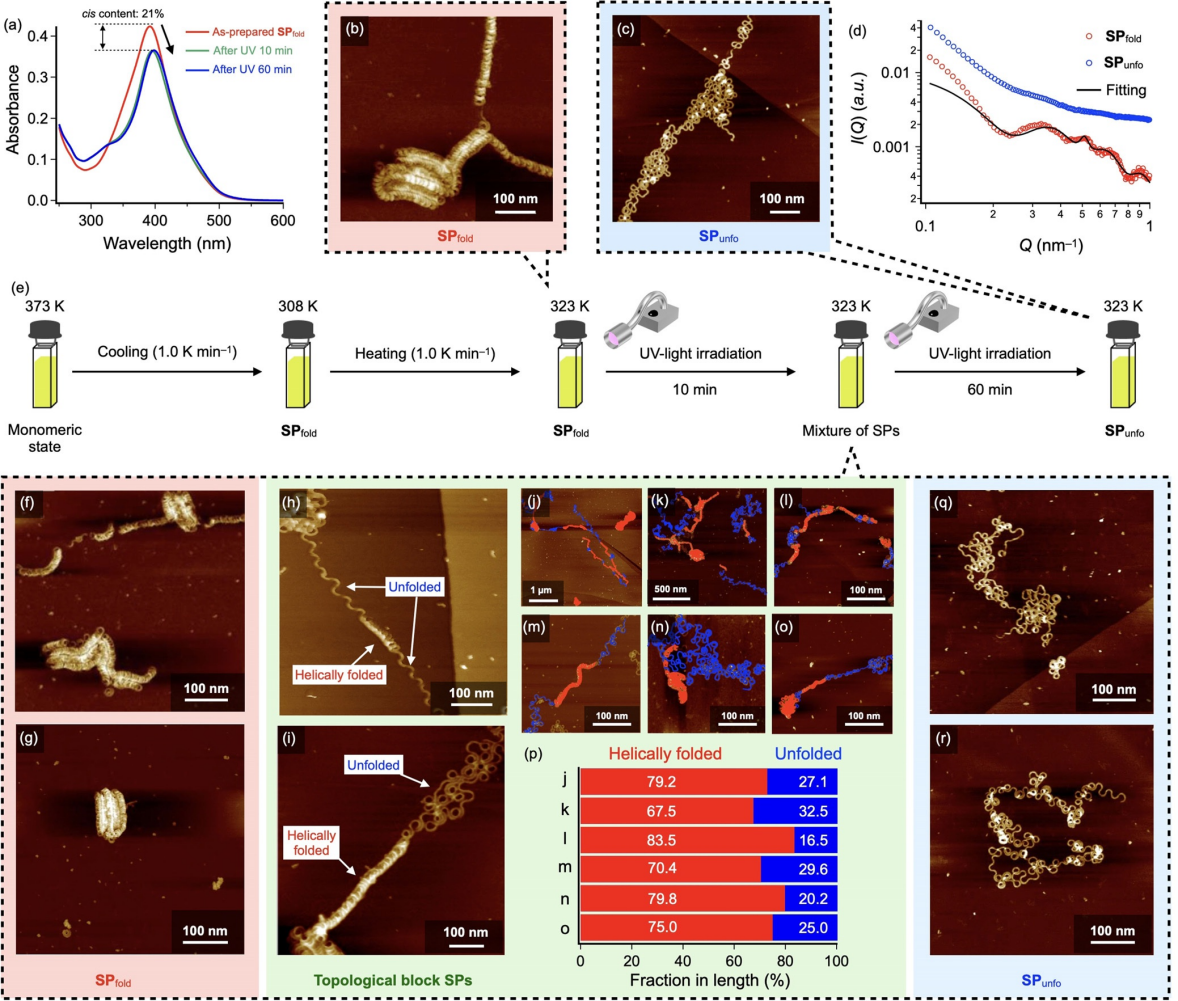
a) UV–Vis spectra of a MCH solution of **2** (*c=*10 μM) during UV‐light irradiation at 323 K. b,c) AFM images of **SP**_fold_ of **2** before UV‐light irradiation (b) and **SP**_unfo_ of **2** after UV‐light irradiation for 60 min at 323 K (c). d) Change of SAXS profiles of a **SP**_fold_ solution of **2** (*c=*50 μM) upon UV‐light irradiation at 323 K (from red to blue). The black line is a fit to the data using a core‐multishell cylinder model. e) Schematic representation of procedure on photo‐induced unfolding of **SP**_fold_ of **2** (f‐o,q,r) AFM images of **SP**_fold_ (f,g), topological block SPs (h‐o), and **SP**_unfo_ (q,r) found in a solution of **2** upon UV‐light irradiation for 10 min at 323 K. In (j‐o), helically folded and unfolded domains were colored with with red and blue, respectively. p) Fractions in length of helically folded and unfolded domains in the topological block SPs.

In order to unfold **SP**_fold_ by light, UV‐light irradiation was attempted at a higher temperature at which unfolding is entropically more favorable process (Figure 3 e). The heating curve in Figure 2 b shows that the dissociation does not occur significantly at 323 K. At this temperature, the absence of thermal unfolding was confirmed by AFM (Figures 3 b and S9). When the **SP**_fold_ solution was irradiated with UV‐light at 323 K, PSS was achieved by an initial 10‐min irradiation, affording 21 % of *cis*‐isomer (blue line in Figure 3 a). AFM observation revealed that the majority of the **SP**_fold_ have been transformed into topological block structures composed of helically folded and unfolded domains although fully folded **SP**_fold_ and completely unfolded **SP**_unfo_ were also observed (Figures 3 f–r). Figures 3 j–o are AFM images of blocky SPs of which folded and unfolded domains were colored with red and blue, respectively (the original AFM images were shown in Figure S10). The fractions (%) in lengths of each domain in the topological block SPs were also provided in Figure 3 p. Non‐uniformity of domain fraction by SPs in combination with the coexistence of intact **SP**_fold_ (Figure 3 f,g) and the fully unfolded **SP**_unfo_ (Figure 3 q,r) strongly suggests that the photoinduced unfolding proceeds cooperatively in individual SP fibers. The results also imply that the SP main chains initially resist unfolding, but they surrender themselves to unfolding once photoinduced deformation occur. Such a cooperative structural transition reflects the internal rigidity of the SP main chain of **2** in comparison with that of **1** showing the uniform unfolding.^[9]^ It is worthy to note that the unfolding of **SP**_fold_ of **2** does not necessarily occur from termini as unfolded domains could be observed between the helically folded domains. We postulated that unfolding could occur also from defected sites,^[16]^ which in our SPs correspond to locally misfolded domains.^[17]^

Prolonged UV‐light irradiation of the topological block SP solution at 323 K for 60 min afforded fully unfolded **SP**_unfo_ as confirmed by dynamic light scattering (DLS), in situ small angle X‐ray scattering (SAXS) and AFM (Figures 3 c,d, S11). DLS measurements before and after the extended UV‐light irradiation revealed that the polydispersity index (PDI) of SPs became larger from 0.284 to 0.373 (Figure S11), suggesting that the transformation from compact to dispersed structures. In the SAXS measurements, the nonperiodic oscillatory features of **SP**_fold_ that is a characteristic of the static intrinsic curvature became unobservable after the UV‐light irradiation (Figure 3 d). Analysis of the SAXS data of the **SP**_fold_, approximating the helically folded fibers as cylindrical objects with three outer shells (1=alkyl, 2=rosette core, 3=alkyl), and a Lorentzian peak function to represent the helical pitch^[18a]^ gave a hollow central radius *r*_core_=7.4±0.1 nm and thicknesses (*δ*) of the three shells as *δ*_1_=1.4 nm, *δ*_2_=4.8 nm, and *δ*_3_=0.3 nm. This is equivalent to *r*_ave_=11.2±0.2 nm, similar to that found by AFM (*r*_ave_=10.4±0.3 nm). The peak position at *Q*=0.51 nm^−1^ equates to a pitch of 12 nm. The X‐ray contrast between the alkyl chains of **2** and the solvent is low, so SAXS cannot easily distinguish the two. This, and solvent penetration,^[18b]^ explains the lower values of *δ*_1_ and *δ*_3_ than expected for a fully stretched dodecyl chain (1.7 nm). The value for *δ*_2_, which equates to the rosette diameter, is in line with dimensions obtained by SAXS for similarly sized rosette‐forming molecules in previous studies.[^18b^, ^18c^] After UV‐light irradiation, the characteristic scattering peaks significantly weakened, indicating deterioration of loop structure. In line with this, AFM visualized that the **SP**_unfo_ lack any trace of helically folded domains. Importantly, unlike to **1**,^[9]^ unfolding up to linearly extended fibers lacking intrinsic curvature was not observed for **2** even after further prolonged UV‐light irradiation. AFM analysis of the **SP**_unfo_ revealed the presence of curvature with *r*_ave_ of 10.6±0.3 nm, which is almost comparable to that of **SP**_fold_ (Figure S7b). This result also reflects the rigid molecular structure of **2**, by which the curvature of SPs becomes persistent to the internal perturbation induced by the photoisomerization of the azobenzene unit.

It is worthy to note that the above transformation from topological block SPs to **SP**_unfo_ proceeded while keeping the constant amount of *cis*‐isomer (21 %) as is evident from no absorption change upon UV‐light irradiation at 323 K (Figure 3 a).Namely, the *trans*→*cis* photoisomerization of the azobenzene units drives the unfolding of the main chain, but the generated *cis*‐isomers are smoothly reconverted thermally to the *trans*‐isomers.

Thermodynamic parameters of the **SP**_fold_ and the photogenerated **SP**_unfo_ were estimated from the thermal dissociation experiments using UV–Vis spectroscopy in order to gain insight of the impact of temperature on the unfolding. The non‐sigmoidal thermal dissociation curves of **SP**_fold_ and **SP**_unfo_ composed of **2**, in which all azobenzenes were *trans*‐isomers, recorded at several concentrations (*c=*10, 15, 20, and 25 μM), could be fitted with a nucleation‐elongation model (Figure S12),^[19]^ and the resulting elongation temperature (*T*_e_) were used to make the modified van't Hoff plot (Figure S13).^[20]^ The changes of standard enthalpy (Δ*H*°), entropy (Δ*S*°), and Gibbs free energy (Δ*G*°) obtained from the plot were summarized in Table 1. Both Δ*H*° and Δ*S*° of **SP**_unfo_ are significantly smaller than those of **SP**_fold_, which is ascribable to the stabilization of helically folded structures by interloop van der Waals interactions between alkyl chains.[^18a^, ^21^] The difference of Δ*G*° values (=Δ*G*° (**SP**_unfo_)−Δ*G*° (**SP**_fold_)) were 10.3 and 2.7 kJ mol^−1^ at 308 and 323 K, respectively. Such large change of Δ*G*° against temperature is because of entropic effect. As described above, the no unfolding proceeded under UV‐light irradiation at 308 K. These results indicate that the entropic effect is also important factor although main driving force of the unfolding is the large structural change of rosettes. Indeed, the photo‐unfolded **SP**_unfo_ spontaneously refolded into **SP**_fold_ upon aging at 308 K for 18 h, supporting the drastic impact of entropic effect (Figure S14).

**Table 1:** Changes of standard enthalpy (Δ*H*°), standard entropy (Δ*S*°), and Gibbs free energy (Δ*G*°) of **SP**_fold_ and **SP**_unfo_ of **2**.

	Δ*H*° [kJ mol^−1^]	Δ*S*° [J mol^−1^ K^−1^]	Δ*G*° at 323 K [kJ mol^−1^]	Δ*G*° at 308 K [kJ mol^−1^]
**SP**_fold_	−114.8	−222.8	−36.1	−46.2
**SP**_unfo_	−53.6	−57.6	−33.3	−35.9

To obtain a molecular‐level insight into the observed inhomogeneous photoinduced unfolding of the SP fiber of **2**, we conducted high‐resolution molecular simulations for polymeric rosette stacks of **1** and **2** in native unperturbed conditions as well as upon transition of the excited azobenzene units. Recently, molecular models of a photo‐responsive supramolecular tubule allowed to observe that the isomerization of the azobenzene units contained in the self‐assembling monomers under UV‐light irradiation occurs and proceeds at defected sites in the tubule structure.^[16a]^ Less ordered domains and defects, which are in principle unavoidably present in soft self‐assembled materials such as SPs, constitute spots where the transitions of excited units are more probable to occur.^[16a]^ The cascade of isomerization that tends to localize in proximity of such less defined domains in the assemblies may then provoke a non‐uniform isomerization of excited groups over time, and a cooperative response by the assembly structure.

**Figure 4:**
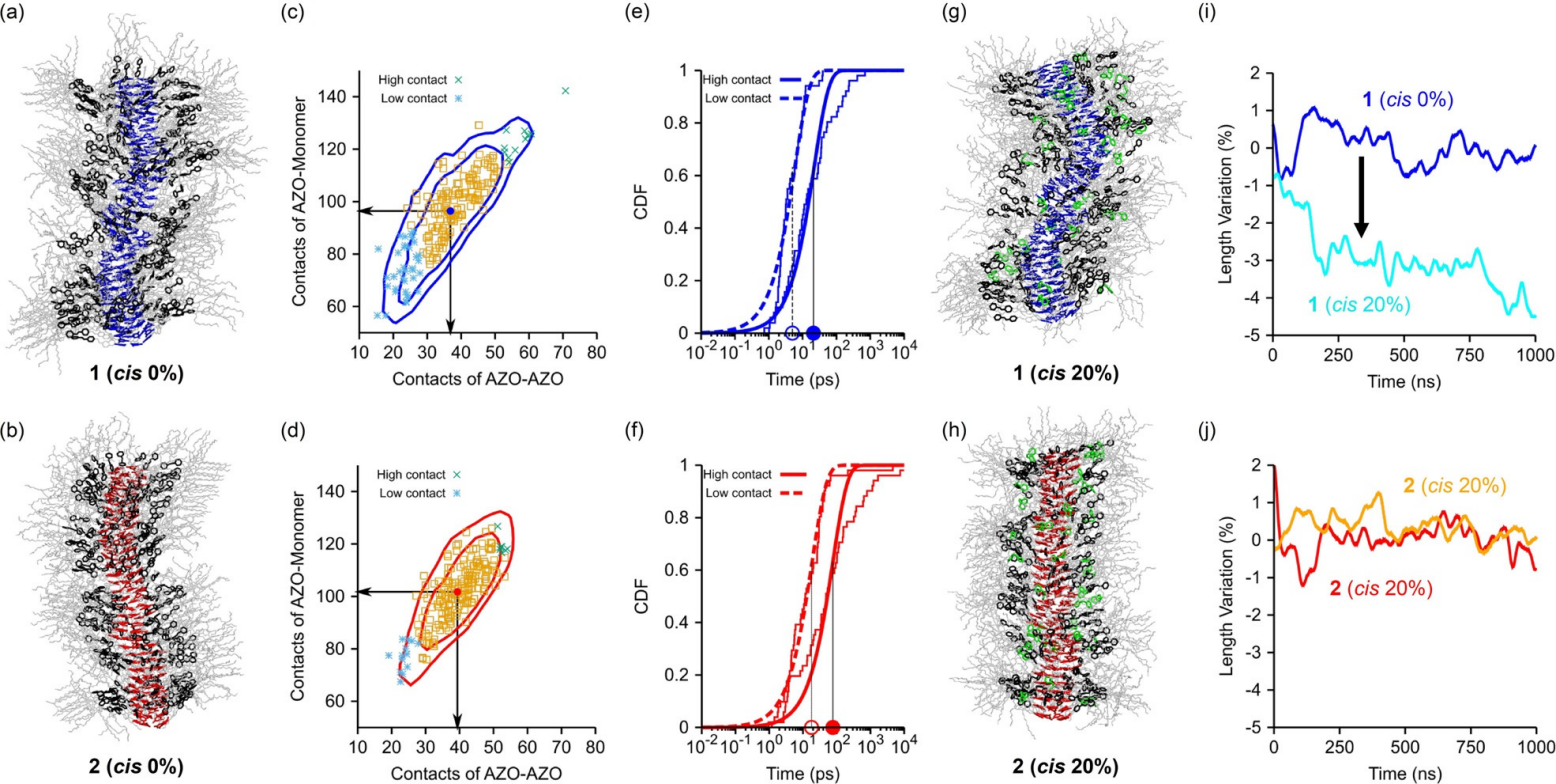
a,b) Equilibrated MD snapshots of **SP1** (a) and **SP2** (b) after 1 μs of MD simulation. Barbiturate groups are colored in blue for **SP1** and in red for **SP2**, respectively, the (*trans*) azobenzene units are colored in black. The rest of the monomers is colored in shaded gray. c,d) Distribution of the azobenzene units based on their interactions/contacts with the surrounding environment: the *x* axis reports the number of contacts of the azobenzenes with the other the azobenzene units in the fibers, while the *y* axis reports the number of contacts between the azobenzene units and the rest of the monomers. The average is indicated by the blue and red points (average contacts values identified by the black lines). Isolines identify those configurations within 0.5 kcal mol^−1^ (inner isolines) and 1.0 kcal mol^−1^ (outer isolines) of free energy penalty from the average (most favorable state) for **SP1** (c) and **SP2** (d). The monomers showing higher number of contacts and lower number of contacts are shown as green and blue points, respectively (the other monomers closer to the average are shown in orange). (e,f) Poissonian fits of the isomerization times for excited azobenzene units with high (solid line) and low contacts (dashed line) in **SP1** (e) and **SP2** (f). From the Poisson fits it is possible to calculate the characteristic timescale for the isomerization in the cases of low and high contacts for **SP1** and **SP2** (black vertical lines intercepting the *x* axis on the characteristic timescales). g,h) Equilibrated structures of **SP1** (g) and **SP2** (h) with 20 % *cis*‐isomerized monomers (*cis* azobenzene in green) after 1 μs of MD simulation. i,j) Variation (in %) of the fiber lengths along the MD simulations calculated respect to the average lengths of the non‐isomerized pre‐equilibrated **SP1** (i) and **SP2** (j).

Here we used fully atomistic molecular dynamics (MD) simulations to obtain insights on the mechanism by which the azobenzene isomerization occurs in the SPs. First, we built atomistic models of SP fibers of **1** (**SP1**) and **2** (**SP2**), composed of 192 monomers initially arranged in 32 perfectly pre‐stacked rosettes. These initially extended fiber models have been then equilibrated via 1 μs of MD simulation in explicit MCH solvent at 297 K (see Supporting Information for details on the models and simulations). From these MD runs, we obtained equilibrated structures of **SP1** and **SP2** (Figures 4 a,b). While the two fibers start from the same configuration, **SP2** was observed to be slightly longer and more persistent at the MD equilibrium than **SP1**, consistent with a higher level of internal rigidity of **SP2**. We analyzed the environment that surrounds the azobenzene units in the two ordered‐domain fibers represented by our models. We calculated the distributions of the azobenzene units in the two equilibrated fiber sections in terms of contacts between them (only azobenzene units) and contacts with the entire surrounding monomers in **SP1** and **SP2** (Figure 4 c,d). The average contact values for the azobenzene units in the two fibers are identified by the black lines in the distribution plots. The slightly lower average values and the broader distribution obtained for **SP1** compared to **SP2** suggests a slightly tighter and more ordered packing of the azobenzene units in **SP2** compared to **SP1**. This can be related to the higher atomic density surrounding the azobenzene units, which is related to their shorter radial distance from the center of the fiber in **SP2**. For such geometrical reasons, in **SP1**, this provokes a slightly reduced stacking between the azobenzene units compared to **SP2**.

Previous studies demonstrated that the degrees of molecular crowding in the environment surrounding the azobenzene units can have an important effect on how the excited chromophores isomerize in the stack: It can occur randomly if the crowding effect is reduced whereas it can occur in spatially localized manner if the crowding effect is dominant.^[16a]^ To obtain indications on how the isomerization most likely accumulate in the stack, we used previously validated atomistic model of excited azobenzenes.^[22]^ We investigated the isomerization of the azobenzene units that are extreme in the obtained azobenzene crowding distributions—i.e., those azobenzene groups which are more loosely (blue points, slightly lower contacts than the average) or more tightly (green points in the distributions, slightly higher contacts than the average) packed in the assembled fibers. The MD simulations showed that the azobenzene units having the highest number of contacts, if excited, have a characteristic isomerization timescale that is just ∼ 5 times slower than that of the azobenzene units with the lowest number of contacts (Figures 4 e,f). The Poisson distributions related to the azobenzene units with higher contacts (Figure 4 e,f: solid curves) are translated on the right (slower kinetics) compared to those of the loosely packed azobenzene units (dashed curves). Black lines in Figures 4 e,f identify the characteristic transition times calculated by the cumulative Poisson fits of azobenzene transitions occurring in such molecular models of ordered helical sections of the SPs. In all cases, the MD simulations provided characteristic transition timescales for excited azobenzene units below the nanosecond scale. In this model of an ordered section of **SP1**, the characteristic isomerization timescale for the excited azobenzene units was found to be in the order of picoseconds, very similar to that expected for free/disassembled azobenzenes in solution.^[22]^ This suggests that in **SP1**, the eventual presence of defects or less ordered domains than the ones present in such ordered models (likely present in the real systems in such soft SP fibers), will have little impact on how the transitions accumulate in the assembly: i.e., in all cases the isomerization would occur randomly along **SP1**. This is consistent with the rather uniform reorganization of **SP1** upon light excitation seen in the experiments. On the other hand, the isomerization in **SP2** occurred with a slower characteristic timescale (i.e., they are less probable) within the ordered section model (Figure 4 e vs. Figure 4 f). This suggests that, in **SP2**, defects or less ordered domains where the azobenzene units are less tightly packed than in these ordered **SP** sections (and where the isomerization may occur faster), constitute spots along these assemblies where the isomerization are more favored to accumulate. This is consistent with the cooperative and non‐uniform reorganization of **SP2** upon light excitation seen in the experiments.

Finally, starting from a preequilibrated structures of the SP fibers (Figure 4 a,b), we also carried out MD simulations where 20 % (experimentally observed value at PSS) of the *trans*‐azobenzene units in **SP1** and **SP2** were converted into *cis*‐isomers (Figure 4 g,h: *cis* isomers in green). Such 20 % *cis*‐SP models were then simulated via MD. Analysis of the global SP structures during the simulations demonstrates that, following to the azobenzene isomerization, the fiber length of **SP1** changed over time (Figure 4 i: cyan vs. blue curves). Conversely, the structure of **SP2** remained substantially unchanged (Figure 4 j: orange vs. red curves). This observation reflects very well the higher rigidity of **SP2**, and provides a rational explanation for the experimentally observed non‐uniform and cooperative UV‐induced unfolding of the **SP**_fold_ of **2**, which afforded topological block SPs.

## Conclusion

We have demonstrated a novel strategy to synthesize topological block supramolecular polymers consisting of two distinct higher‐order structures, helically folded and unfolded domains. Our strategy is based on post‐polymerization photoinduced unfolding of helically folded supramolecular nanofibers consisting of six‐membered hydrogen‐bonded rosettes of barbiturates with azobenzene photoswitches. To achieve non‐uniform unfolding of helically folded structures upon isomerization of internal azobenzene units, the previously designed flexible monomer structure was modified to a more robust structure, by which supramolecular nanofibers became more curvature‐persistent toward azobenzene isomerization. This monomer‐based improvement in the robustness of supramolecular polymer chains has been reproduced by MD simulations of fibers consisting of the previous flexible and the current robust monomers upon isomerization of their azobenzene units. The improved robustness of supramolecular polymer chains realized non‐uniform unfolding of helically folded structures into fully unfolded structures through intermediate topological block structures. Owing to the improved curvature‐persistency, spontaneous re‐folding of the fully unfolded structures has been observed unlike the previous system. Our study thus demonstrates unprecedented nanofabrication of one‐dimensional nanomaterials through the post‐self‐assembly partial transformation of their structures. We believe that the present study will motivate researchers to develop more precise nanofabrication of supramolecular soft materials.

## Conflict of interest

The authors declare no conflict of interest.

## Supporting information

As a service to our authors and readers, this journal provides supporting information supplied by the authors. Such materials are peer reviewed and may be re‐organized for online delivery, but are not copy‐edited or typeset. Technical support issues arising from supporting information (other than missing files) should be addressed to the authors.

Supporting Information
