# Supplementary material for: Non‐uniform Photoinduced Unfolding of Supramolecular Polymers Leading to Topological Block Nanofibers
Source: Angew Chem Int Ed Engl. 2021 Nov 22;60(52):26986–93. doi: 10.1002/anie.202110224 (PMC9298767; doi:10.1002/anie.202110224)
Supplement: Supplementary file 1 — Supporting Information [file ANIE-60-26986-s001.pdf]

Angewandte Chemie
Eine Zeitschrift der Gesellschaft Deutscher Chemiker

Supporting Information

Non-uniform Photoinduced Unfolding of Supramolecular Polymers Leading to Topological Block Nanofibers

Keigo Tashiro, Kosuke Katayama, Kenta Tamaki, Luca Pesce, Nobutaka Shimizu, Hideaki Takagi, Rie Haruki, Martin J. Hollamby, Giovanni M. Pavan, and Shiki Yagai**

anie_202110224_sm_miscellaneous_information.pdf

Table of Contents

Supporting method

Materials

Nuclear magnetic resonance (NMR) spectroscopy, electrospray ionization mass (ESI-MS) and atmospheric pressure chemical ionization mass (APCI-MS)

UV/vis spectroscopy

Atomic force microscopy (AFM)

Dynamic light scattering (DLS)

Estimation of degree of aggregation (α_{agg})

Cooperative (nucleation-elongation) model fitting

Small-Angle X-Ray Scattering

SAXS Analysis

Molecular dynamic (MD) calculation

Synthesis

Compound **3**

Compound **4**

Compound **2**

Photoisomerization of monomeric 2

Results and Discussion

Supporting figures

References

Supporting methods

Materials

Compound **2** was synthesized by following procedures written below. All reagents were purchased from commercial suppliers and utilized without further purification. Column chromatography was performed using 63–210 μm silica gel. The solvents for the preparation of assemblies were all spectral grade and used without further purification.

Nuclear magnetic resonance (NMR) spectroscopy, electrospray ionization mass (ESI-MS) and atmospheric pressure chemical ionization mass (APCI-MS)

NMR spectra of ^{1}H and ^{13}C were recorded on BRUKER AVANCE III-400M and JEOL JMN-ECA500 NMR spectrometer at 400 and 500 MHz, respectively. ^{1}H NMR chemical shift in ppm (δ) were normalized with the chemical shift of tetramethylsilane at 0.00 ppm. The resonance multiplicity was represented in terms of s (singlet), d (doublet), m (multiplet), br (broad), and brs (broad singlet). ^{13}C NMR chemical shifts reported in ppm (δ) were normalized with the chemical shifts of $CDCl_3$ at 77.16 ppm. ESI-MS and APCI-MS spectra measurements were conducted on an Exactive (Thermo Scientific).

UV/vis spectroscopy

UV/vis absorption spectra were measured by JASCO V660 spectrophotometer equipped with a JASCO ETCS-761 temperature controller. Supramolecular polymers in methylcyclohexane (MCH) were added into a quartz cell with screw cap and optical path length was 1.0 cm.

Atomic force microscopy (AFM)

AFM imaging was carried out under ambient conditions using Multimode 8 Nanoscope V (Bruker Instruments) in Peak Force Tapping (ScanAsyst) mode. Silicon cantilevers (SCANASYST-AIR) with a spring constant of 0.4 N/m and frequency of 70 kHz (nominal value, Bruker, Japan) were employed. MCH solution of supramolecular polymer (10 µL) was spin-coated (3000 rpm, for 1 min) onto freshly cleaved highly oriented pyrolytic graphite (HOPG) substrate. AFM images were processed using NanoScope Analysis 1.40 (Bruker).

Dynamic light scattering (DLS)

DLS measurements were performed on a Zetasizer Nano S (Malvern Instruments) device using non-invasive back-scatter technology (NIBS) with He-Ne laser (633 nm, 4.0 mW). The scattering angle was set at 173°.

Estimation of degree of aggregation (α_{agg})

For the temperature-dependent UV/vis measurements, the degree of aggregation (α_{agg}) was calculated from Supplementary Equation S1:

$$\alpha_{\mathrm{agg}} = \frac{\mathrm{Abs}(T) - \mathrm{Abs}_{\mathrm{mon}}}{\mathrm{Abs}_{\mathrm{agg}} - \mathrm{Abs}_{\mathrm{mon}}} \tag{S1}$$

where Abs_{agg} and Abs_{mon} are absorbances at 465 nm of aggregated (the highest value) and pure monomeric species (the lowest value), respectively, and Abs(T) is the absorbance at 465 nm on a given temperature.

Cooperative (nucleation-elongation) model fitting

Cooperative assembly processes were analyzed by a nucleation-elongation model proposed by Meijer and co-workers.[1] When the plot of degree of aggregation (α_{agg}) versus T is non-sigmoidal, the polymerization process can be followed by cooperative model.

In the elongation regimes ($T < T_e$) were fitted with Supplementary Equation S2:

$$\alpha_{\mathrm{agg}} = \alpha_{\mathrm{SAT}} \left[1 - exp\left(-\frac{\Delta H_{\mathrm{e}}}{\mathrm{R}T_{\mathrm{e}}{}^{2}} (T - T_{\mathrm{e}}) \right) \right] \tag{S2}$$

where ΔH_e is the enthalpy release in the elongation regime, α_{SAT} is a parameter the correction coefficient, R is the ideal gas constant, and T_e is critical temperature.

In the nucleation regimes ($T > T_e$) were fitted to Supplementary Equation S3:

$$\alpha_{\mathrm{agg}} = \alpha_{\mathrm{SAT}} \cdot \sqrt[3]{\mathrm{K_a}} \cdot exp\left[\left(\frac{1}{3\sqrt[3]{K_{\mathrm{a}}}} - 1 \right) \frac{\Delta H_{\mathrm{e}}}{\mathrm{R}T_{\mathrm{e}}{}^{2}} (T - T_{\mathrm{e}}) \right] \tag{S3}$$

where K_a is the dimensionless activation equilibrium constant at T_e and is defined as $K_a = K_n/K_e$, which K_n and K_e is the equilibrium constant of nucleation and elongation, respectively. The nucleation step is governed by K_a, a parameter reflecting degree of cooperativity.

Small-Angle X-ray Scattering (SAXS)

SAXS experiments were performed at BL-10C of the Photon Factory of the High Energy Accelerator Research Organization (KEK) in Tsukuba, Japan48. Sample solutions were placed into 1.25-mm path length cells (20-μm thickness quartz glass windows surrounded by stainless steel), and the temperature was fixed at 293 K. X-ray with a wavelength of 1.5 Å and a sample-detector distance of 1029mm (calibrated with silver behenate) resulted in a detectable *Q* range in the order of 0.1–5.9 nm^{-1}. Sixty frames were collected with each exposure time of 10 s. Because no radiation damage was observed, the collected data were averaged to give a total integration time of 600 s. The 2D scattering data (detector: DECTRIS PILATUS3 2M) were radially averaged to yield 1D scattering intensity data [*I*(*Q*) vs. *Q*]. These data were then normalized using water as a reference, and the following subtraction of the background (quartz glass windows and solvent) gave scattering intensity *I*(*Q*). All data reduction was performed using the software package SAngler49. SAXS data were collected using X-ray with a wavelength of 1.5 Å

SAXS Analysis

SAXS data analysis was carried out using SasView (https://www.sasview.org/). Data arising from the helically folded structure was approximated by a core-multishell cylinder (Figure S1), with the cylinder length being the z-direction (direction of the helicoidal pitch). An additional Lorentzian peak function was used to describe the peak arising from the pitch.[3] The core is then comprised of solvent, and the three shells are then (1) alkyl chains within the rosette that pointing into the core of the helically folded structure (2) the central, more electron dense rosette core and (3) alkyl chains within the rosette that point away from the helically folded structure. While still an approximation of the structure (not taking into account the 3D nature of the strands, nor the space between them and not allowing for flexibility in the helically folded structure) this is a more sophisticated model than has been previously used[4] and allows the complex internal sld profile of the helically folded strand to be at least partially recreated. It provides a far more accurate agreement with the data in the mid- high *Q* region than could be obtained using previously applied approximated models (*e.g.*, representing the helically folded structure as a core + single shell cylinder, or as toroid). [4]

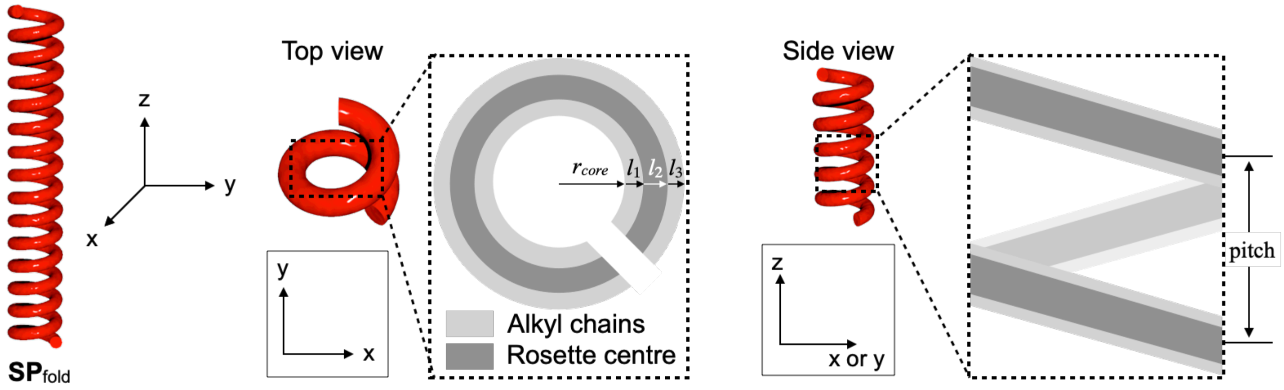

Figure S1. Schematic depiction of helically folded structure on each sight. The core and 3 shells (Top view) were approximated by the core-multishell cylinder model.

Within the core + three shell cylinder, the overall scattered intensity is calculated using Supplementary Equation S4.

$$I(q,\alpha) = \frac{scale(cyl)}{V_{total}} F^2(q,\alpha).\sin(\alpha) + I_{peak} + I_{bkg} \quad \text{(S4)}$$

In the above, α is the angle between the cylinder axis and the scattering vector, q. V_{total} is the total cylinder volume, including shells and the scale(cyl) is related to the volume fraction. F(q,α) is given as follows:

$$F(q,a) = \sum_{k=0}^{3} \left[(\rho_k - \rho_{k+1}) V_k \frac{\sin(1/2\, L.q.\ \cos\alpha)}{1/2\, L.q.\ \cos\alpha} \frac{2J_1(q.r_k \sin\alpha)}{q.r_k \sin\alpha} \right] \exp\left\{ -\frac{1}{2} q^2 \sigma^2 \right\} \quad \text{(S5)}$$

In Supplementary Equation S5, $V_k = \pi L_{core}\, r_k^2$ is the volume of each shell k, with $r_k = r_{k-1} + l_k$, where $r_{k=0}$ = r_{core} (core radius) and l_k is the thickness of shell k. ρ_k is the scattering length density (of shell "k". The core radius lies when k = 0. Here, $\rho_k = \rho_0$ and therefore $\rho_0 = \rho_{solvent}$ = 7.45 × 10^{-6} Å^{-2}. Likewise, when k = 3, $\rho_{k+1} = \rho_{solvent}$ = 7.45 × 10^{-6} Å^{-2}. For the alkyl shells (k = 1 and 3), ρ = 7.3 × 10^{-6} Å^{-2} was used. In considering the scattering length density of the 2nd shell, which includes contributions from the rosette centres it is important to note that while $\rho_{rosette\_core}$ ~ 12 × 10^{-6} Å^{-2} when considering the helically folded structure as a cylinder, the strands comprising stacked rosettes (and their cores) do not occupy the whole shell region in the z direction, which also includes alkyl chains and a significant amount of solvent. When allowed to float, $\rho_{k=2}$ tended to adopt lower values and was heavily correlated to other parameters, and so was fixed at $\rho_{k=2}$ = 8.5 × 10^{-6} Å^{-2}, which was calculated on the basis of the position of the pitch peak and the modelled shell thicknesses, additional allowing a slight ellipsoidal cross section from tilted packing as observed in previous combined SAXS and SANS studies of related systems.

Finally, the Lorentzian peak function is given by Supplementary Equation S6, in which scale(peak) is a scale factor, q_0 is the peak position in q, and B is the half-width at half-maximum.

$$I_{\text{peak}} = \frac{scale(peak)}{\left(1 + \left(\frac{q - q_0}{B}\right)^2\right)} \quad \text{(S6)}$$

The quality of the fit to the data is shown in Figure 3d in the manuscript. As can be seen, in the mid- and high-Q regions the fit is of high quality, but it fails to explain the shape of the data at lowest Q. The fitted parameters obtained using the model outlined in the Supplementary Equation S4 to the data using the SasView software are provided in Table S1. As noted in the MS, the radius and length parameters yield a strand centre–to–strand centre diameter [approximated here as $2(r_{core} + l_1 + ½l_2)$] of 11.3 nm, which compares well with the value obtained by analysis of AFM images. By performing repeated analyses, in particular looking at different values for $\rho_{k=2}$, the error on r_{core} was found to be relatively low (± 0.5 nm), but errors on dimensions $l_{1\text{-}3}$ were proportionally higher, and for l_1 and l_3 were further compounded by the poor SAXS contrast between the alkyl chains and solvent background.

Finally, the value of *L* is lower than might be expected given the noted length of the helically folded structures by AFM. As noted above, while sophisticated the model applied here is still only a very primitive explanation for the 3-dimensional structure of these complex hierarchical assemblies. Fixing *L* was attempted, but this led to poorer agreement throughout the *Q* range. It is possible therefore that the fitted value of *L* might be an indication of the distance over which the structure remains rigid, but the model has no means to account for the aggregation of several cylinders into a longer chain, hence the poor agreement with the data at low *Q*.

Table S1. Parameters obtained through analysis of SAXS data using the model described above.

scale(cyl)	0.38
r_{core} / nm	6.8
L / nm	3.0
scale(peak)	6.2×10^{-4}
q_0 / nm^{-1}	0.51
B	0.031
l_1 / nm	2.5
l_2 / nm	4.1
l_3 / nm	1.4
I_{bkg}	7.6×10^{-5}

Molecular dynamic (MD) calculation

The entire system was studied computationally at the atomistic level, in order to maximize the resolution of the system while keeping the possibility of inquiring the supramolecular aspects of the assemblies. The **1** and **2** monomers and methylcyclohexane were parametrized based on the General Amber Force Field, (GAFF).[5] The partial charges for the monomer atoms were calculated with the AM1-BCC method[6] using the antechamber module of AmberTools.[7] The detailed parametrization procedure and the corresponding output files are published on the Zenodo data repository.[8]

All the simulation herein performed, were run on GROMACS-2020.5[9] patched with plumed-2.7.[10] All systems were simulated for 1 μs of MD at the temperature of 297 K and pressure of 1 atm in explicit methylcyclohexane solvent in periodic boundary NPT conditions (constant N: number of particles, P: pressure and T: temperature), employing the v-rescale thermostat[11] and the isotropic Berendsen barostat.[12] A timestep of 2 fs was used in the MD simulations. The electrostatic interactions were treated using particle mesh Ewald (PME).[13] The cutoff lengths of the real summation and of the VdW were set to 1.0 nm. The dynamics of the hydrogens was constrained using the LINCS algorithm.[14]

The assemblies were pre-assembled in order to form an initially perfect and extended tube (aligned to the *z*-axis of the box) of 32 rosettes each containing 6 monomers. This conformation allows each monomer to from 4 hydrogen bonds with the neighbor monomers belonging to the same rosette. In order to pre-equilibrate solvophilic-domain of the structure, after minimization of the whole system, the barbiturate's heavy atoms were restrained for 10 ns MD run. After this, another run of minimization was performed. The following equilibration and production MD runs were performed restraining the barbiturate heavy atoms to move along the *z*-axis for the six monomers at the top and at the bottom of the tube. In this way it was possible to the structure

to readjust its structure without losing the integrity of the tips. These atomistic models of ordered sections of **SP1** and **SP2** prior to isomerization have been equilibrated for 1 μs of MD. From these MD runs, we could estimate the evolution of the length of the SP models along the MD, as well as the contacts between the azobenzene units within the ordered **SP** sections.

For the study of the photoisomerization of the azobenzene moieties of the **1** and **2** monomers, 36 monomers randomly placed along the ordered bulk of the fiber modeled sections were chosen (excluding the monomers from the tips). The isomerized 20% of *cis* groups were inserted into pre-equilibrated **SP1** and **SP2** (after ~100ns of equilibration MD). From these starting points, additional 1μs of MD allowed us to gather information on the isomerization timescales collected in Figure 4e-f. Short MD runs of these ordered tubes section models allowed then to estimate more precisely the isomerization timescales that can be expected for the monomers having highest and lowest contacts within the ordered sections of these **SP**s. All the raw data from the simulations and the related analyses are available in the Zenodo data repository.[8]

Statistical length analysis of folded/unfolded domain in topological block SPs

Statistical length analysis was carried out by estimating fractions of folded and unfolded domains in length in the topological block SPs. The length of folded domain (L_{fold}) was calculated from average radius of curvature (r_{ave}) estimated from AFM images, and the number of loops (n_{loop}) by following Supplementary Equation S7.

$$L_{fold} = 2\pi r_{ave} n_{loop} \tag{S7}$$

On the other hand, the length of unfolded domain (L_{unfo}) was obtained from the image analysis using "imageJ" software. Unfolded domains are manually traced, and then the total length of the traced region was converted to nanometer based on the scale of the AFM image. The fraction in length of folded (x_{fold}) and unfolded (x_{unfo}) domains were calculated according to Supplementary Equations S8 and S9.

$$x_{fold} = \frac{L_{fold}}{L_{fold} + L_{unfo}} \tag{S8}$$

$$x_{unfo} = 1 - x_{fold} \tag{S9}$$

Synthesis

Compound **2** was synthesized according to Scheme S1.

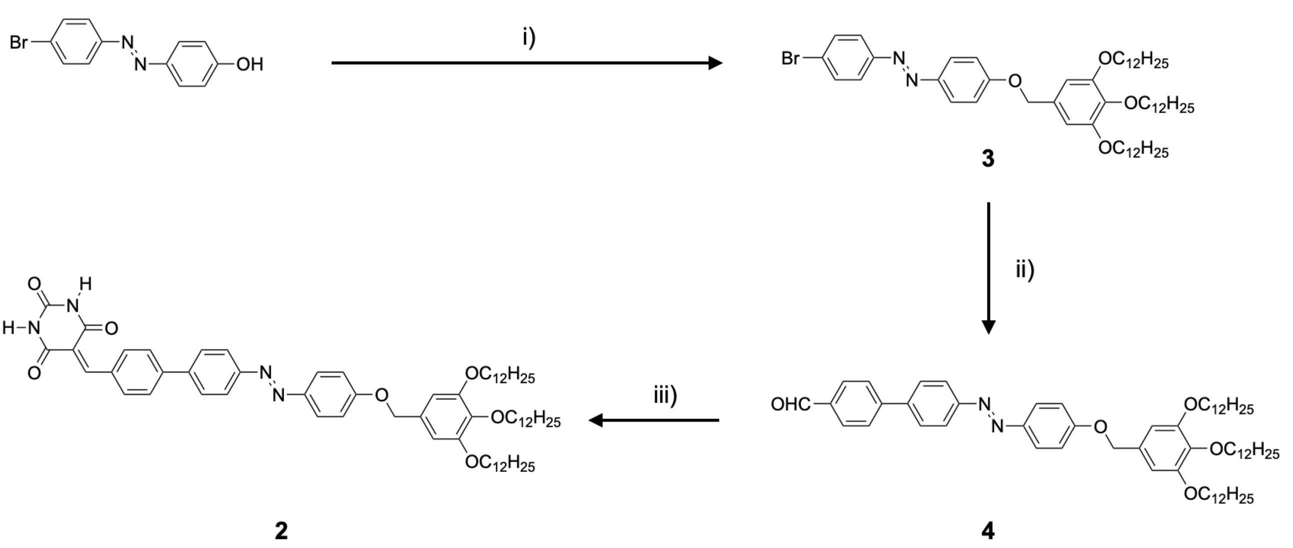

Scheme S1. Synthetic root of **2**. i) 3,4,5-tri(*n*-dodecyloxy)benzyl chloride, K_2CO_3, dry *N*,*N*-dimethylformamide (DMF), 75 °C, 3.5 h; ii) 4-(4,4,5,5-tetramethyl-1,3,2-dioxaborolan-2-yl)benzaldehyde, Pd(pph$_3$)$_4$, K_2CO_3, DMF, 80 °C, 3 h; iii) barbituric acid, ethanol, 85 °C, 9 h. (*E*)-4-((4-bromophenyl)diazenyl)phenol was synthesized by following reference.[2]

(*E*)-1-(4-bromophenyl)-2-(4-((3,4,5-tris(dodecyloxy)benzyl)oxy)phenyl)diazene (3)

(*E*)-4-((4-bromophenyl)diazenyl)phenol (250 mg, 0.88 mmol) was dissolved in a suspension of K_2CO_3 (259 mg, 1.81 mmol) in dry DMF (10 mL) at 75 °C, and mixture was stirred until it became homogeneous. 3,4,5-Tri(n-dodecyloxy)benzyl chloride (600 mg, 0.88 mmol) was added into the solution and the mixture was refluxed at 75 °C for 12 h with stirring. The resulting mixture was diluted with solvent mixture of *n*-hexane and ethylacetate (4:1, v/v) and washed with water and brine. The organic layer was separated, and dried over Na_2SO_4 and the solvent was removed by evaporation. The resulting residue was purified by column chromatography on silica gel (eluent: $CHCl_3$:*n*-hexane = 1:1, v/v) and the orange fraction was collected. The fraction was evaporated to give oily material, which was dried in vacuo to give compound **3** as an orange solid (524 mg, 64.6%). ^{1}H NMR (400 MHz, $CDCl_3$): δ = 7.91 (d, *J* = 9.0 Hz, 2H), 7.76 (d, *J* = 8.8 Hz, 2H), 7.62 (d, *J* = 8.7 Hz, 2H), 7.08 (d, *J* = 9.0 Hz, 2H), 6.63 (s, 2H), 5.04 (s, 2H), 4.00–3.94 (m, 6H), 1.83–1.71 (m, 6H), 1.46–1.26 (m, 54H), 0.89–0.86 (m, 9H); ^{13}C NMR(400 MHz, $CDCl_3$): δ = 161.53, 153.41, 151.50, 147.00, 138.21, 132.25, 131.22, 124.91, 124.63, 124.11, 115.19, 76.72, 73.73, 70.73, 69.21, 31.96, 30.38, 29.78, 29.73, 29.68, 29.45, 29.40, 26.17, 26.13, 22.72, 14.13; MS (ESI): m/z calcd for $C_{55}H_{88}O_4N_2Br$ = 919.5922 $[M+H]^+$, found 919.5934.

(*E*)-4'-((4-((3,4,5-tris(dodecyloxy)benzyl)oxy)phenyl)diazenyl)-[1,1'-biphenyl]-4-carbaldehyde (4)

Compound **3** (1.35 g, 1.47 mmol) was dissolved into a suspension of K_2CO_3 (1.01 g, 7.31 mmol) in dry DMF (20 mL). 4-(4,4,5,5-Tetramethyl-1,2,3-dioxaborolan-2-yl)benzaldehyde (273 mg, 1.91 mmol) and $Pd(PPh_3)_4$ (84.8 mg, 0.073 mmol) was added to the suspension and the mixture was stirred for 3 h at 80 °C. DMF was removed by evaporation, and resultant solid was dried in *vacuo*. The residue was purified by column chromatography on silica gel using toluene as eluent to give compound **4** as an orange solid (390 mg, 28.9%). ^{1}H NMR (400 MHz, $CDCl_3$); δ = 10.08 (s, 1H), 8.00–7.94 (m, 6H), 7.84–7.77 (m, 4H), 7.10 (d, *J* = 9.0 Hz, 2H), 6.64 (s, 2H), 5.05 (s, 2H), 4.00–3.94 (m, 6H), 1.83–1.73 (m, 6H), 1.47–1.26 (m, 54H), 0.89–0.86 (m, 9H); ^{13}C NMR(100 MHz, $CDCl_3$): δ = 191.80, 161.51, 153.41, 152.61, 147.24 146.24, 141.41, 138.21, 135.52, 131.24, 130.33 128.08, 127.72, 124.93, 123.28, 115.20, 106.24, 76.71, 70.74, 69.22, 31.97, 31.95, 30.37, 29.78, 29.72, 29.67, 29.45, 29.41 29.38, 26.12, 22.71, 14.12; MS (ESI) : m/z calcd for $C_{62}H_{92}O_5N_2$ = 945.7079 [M+H]$^+$, found 945.7070.

(*E*)-5-((4'-((4-((3,4,5-tris(dodecyloxy)benzyl)oxy)phenyl)diazenyl)-[1,1'-biphenyl]-4-yl)methylene)pyrimidine-2,4,6(1*H*,3*H*,5*H*)-trione (2)

A mixture of **4** (203 mg, 0.212 mmol) and barbituric acid (270 mg, 2.12 mmol) in ethanol (20 mL) was refluxed for 9 h at 85 °C under N_2 atmosphere. The reaction mixture was cooled to r.t. and the resulting precipitates were collected by filtration. The residue was repeatedly washed with hot ethanol to give pure **2** as an orange solid (157 mg, 70.4%). ^{1}H NMR (500 MHz, $CDCl_3$); δ = 8.61 (s, 1H), 8.32 (d, *J* = 8.2 Hz, 2H), 7.99–7.93 (m, 5H), 7.81−7.79 (m, 5H), 7.10 (d, *J* = 8.8 Hz, 2H), 6.64 (s, 2H), 5.05 (s, 2H), 4.01−3.96 (m, 6H), 1.83−1.74 (m, 6H), 1.48−1.28 (m, 54H), 0.90–0.87 (m, 9H); ^{13}C NMR(125 MHz, $CDCl_3$); δ = 153.53, 135.58, 128.00, 127.06, 124.96, 123.36, 119.20, 115.35, 106.66, 76.77, 73.55, 70.85, 69.51, 31.98, 30.46, 29.80, 29.75, 29.70, 29.58, 29.49, 29.42, 29.40, 26.23, 26.20, 22.71, 14.08; MS (APCI) : m/z calcd for $C_{66}H_{95}O_7N_4$ = 1055.7195 [M+H]$^+$, found 1055.7201.

Photoisomerization of monomeric 2

Photoisomerization of monomeric **2** in $CDCl_3$ was investigated by ^{1}H NMR and UV/vis spectroscopic measurements. The ^{1}H NMR measurement reveal that new peaks at δ = 4.90 ppm (marked with green) attributed to the benzylic protons of the *cis*-isomer appeared after UV-light irradiation to the $CDCl_3$ solution of **2** (99.9% *trans*-isomer, *c* = 1 mM) for 10 min (Figure S8b,c). Calculation of fraction of the *cis* protons (Supplementary Equation S10) indicated a percentage of *cis*-isomer was 40.2%. UV/vis spectroscopic measurement of the $CDCl_3$ solution including **2** (*c* = 10 μM) was also performed (Figure S8d). The UV/vis band at 400 nm attributed to π–π* transition of *trans*-isomer decreased and an increase of absorption peak around 500 nm arising from n–π* transition of *cis*-isomer was concurrently observed after UV-light irradiation for 10 min. It should be noted that no spectral change was found upon further UV-light irradiation, indicating attainment of a photo-stationary state (PSS). When *trans*-isomer reached PSS, 19.2% of the absorbance at 400 nm diminished. However, the percentage of the *cis*-isomer at PSS was calculated as 40.2% from the ^{1}H NMR measurement, the degree of the isomerization obtained from absorption spectra was almost half. This result might be derived from overlap between absorption peaks of azobenzene and biphenyl moieties in **2**. From these results, we can presume that **2** reaches to PSS when the absorbance around 400 nm exhibits ca. 20% of decrease.

$$cis\ content\ (\%) = \frac{I\,(\delta = 4.90\ \text{ppm})}{I\,(\delta = 4.90\ \text{ppm}) + I\,(\delta = 5.05\ \text{ppm})} \times 100 \qquad \text{(S10)}$$

where, I (δ = 4.90 ppm) and I (δ = 5.05 ppm) are integral intensity of the protons at benzylic position in *cis*- and *trans*-isomers marked with red and bule, respectively, as shown in Figure S8a.

Results and Discussion

Supporting figures

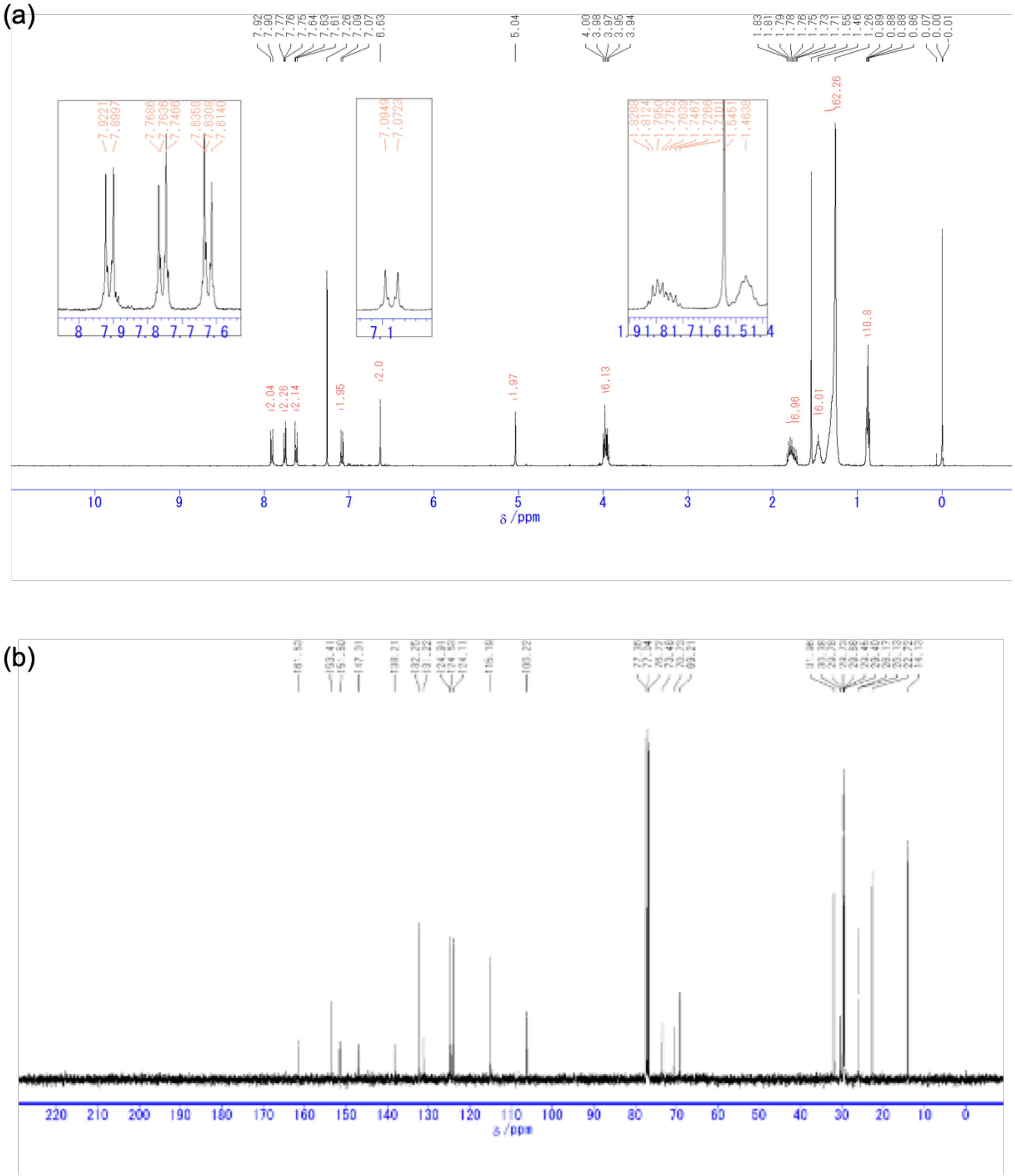

Figure S2. (a) ^{1}H NMR and (b) ^{13}C NMR of **3** in $CDCl_3$ at 298 K.

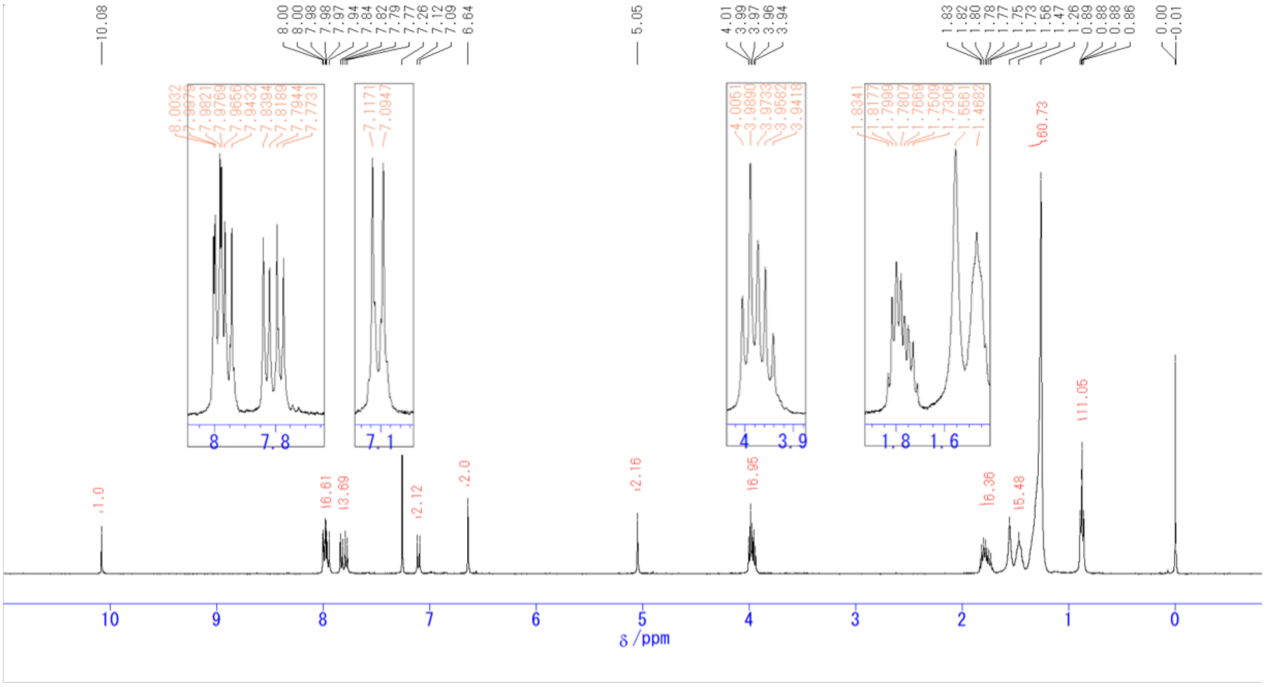

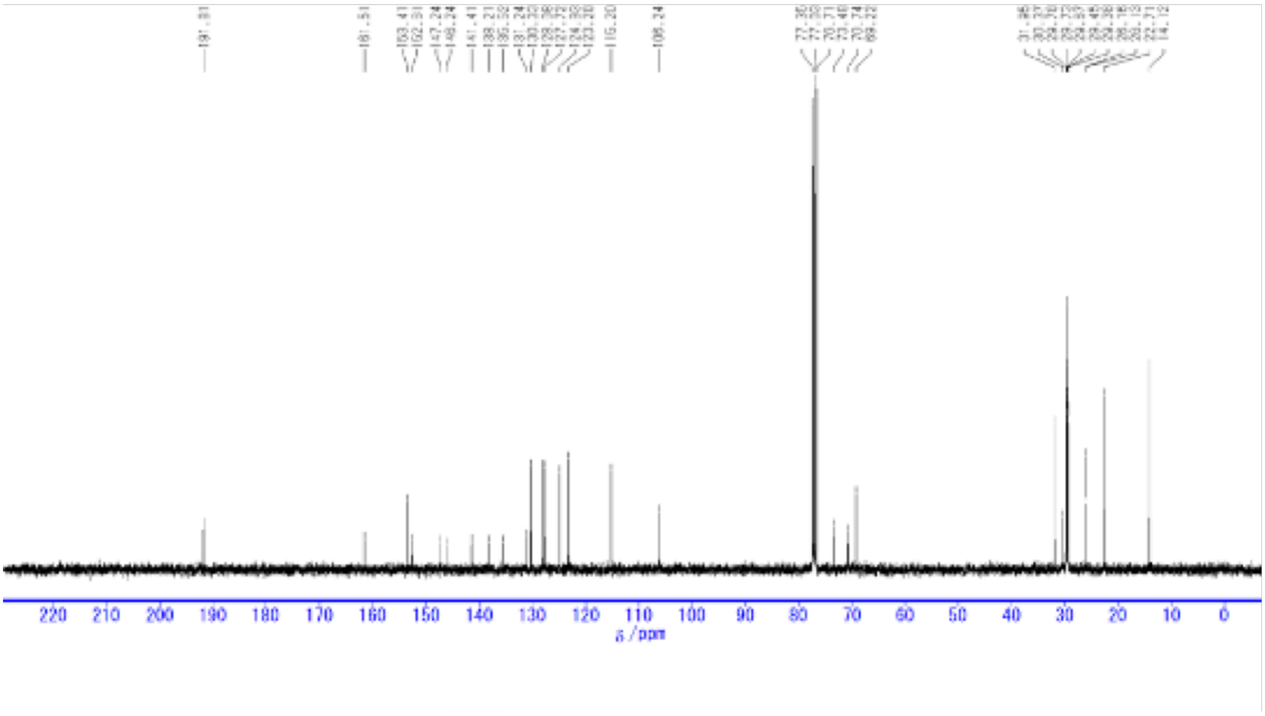

Figure S3. (a) ^{1}H NMR and (b) ^{13}C NMR of **4** in $CDCl_3$ at 298 K.

(a)

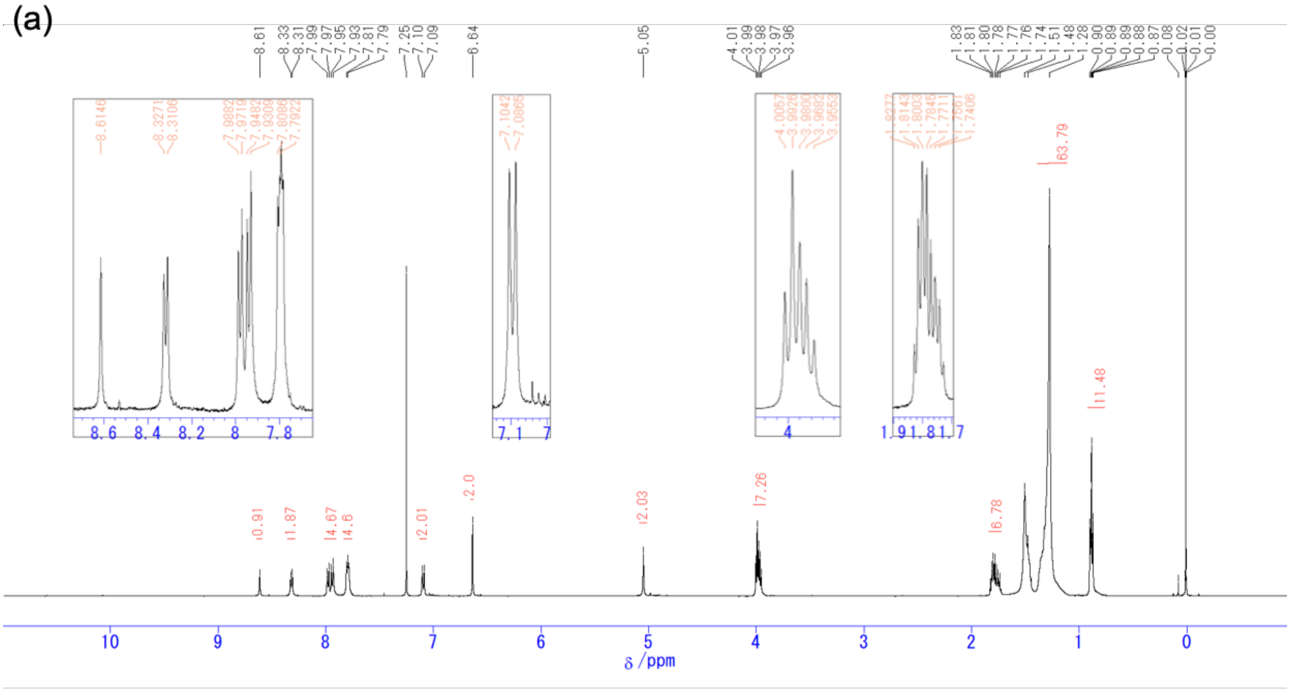

(b)

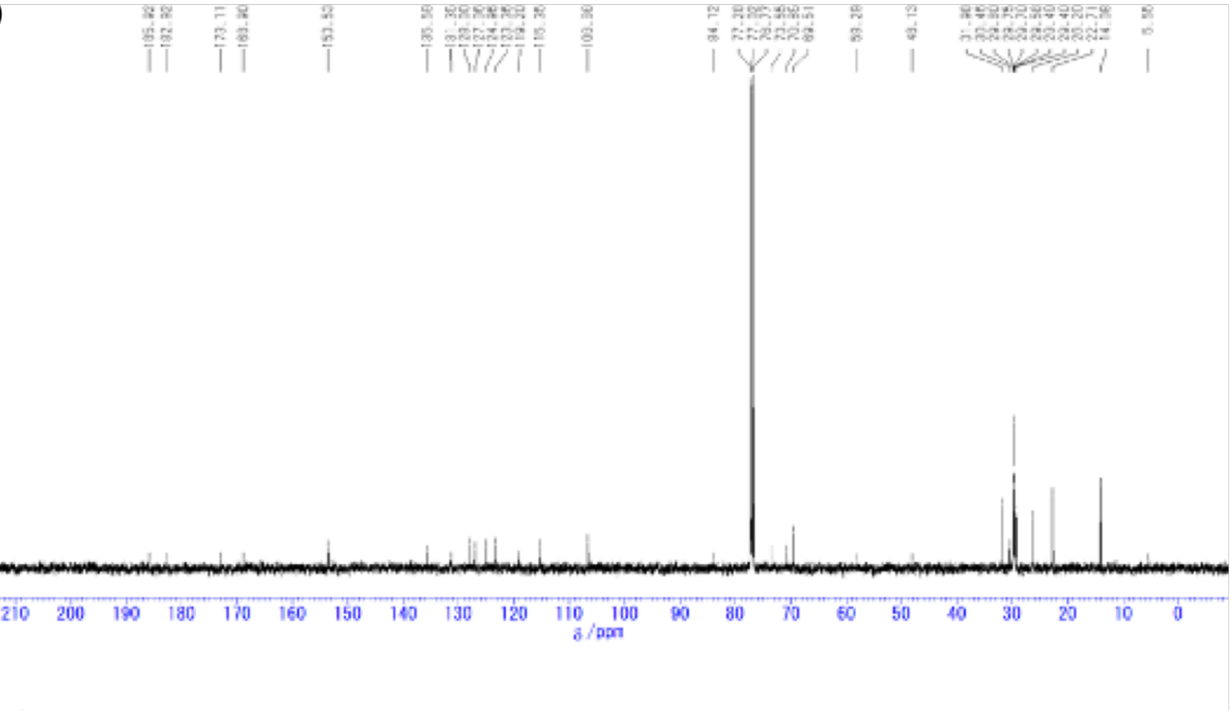

Figure S4. (a) ^{1}H NMR and (b) ^{13}C NMR of **2** in $CDCl_3$ at 323 K.

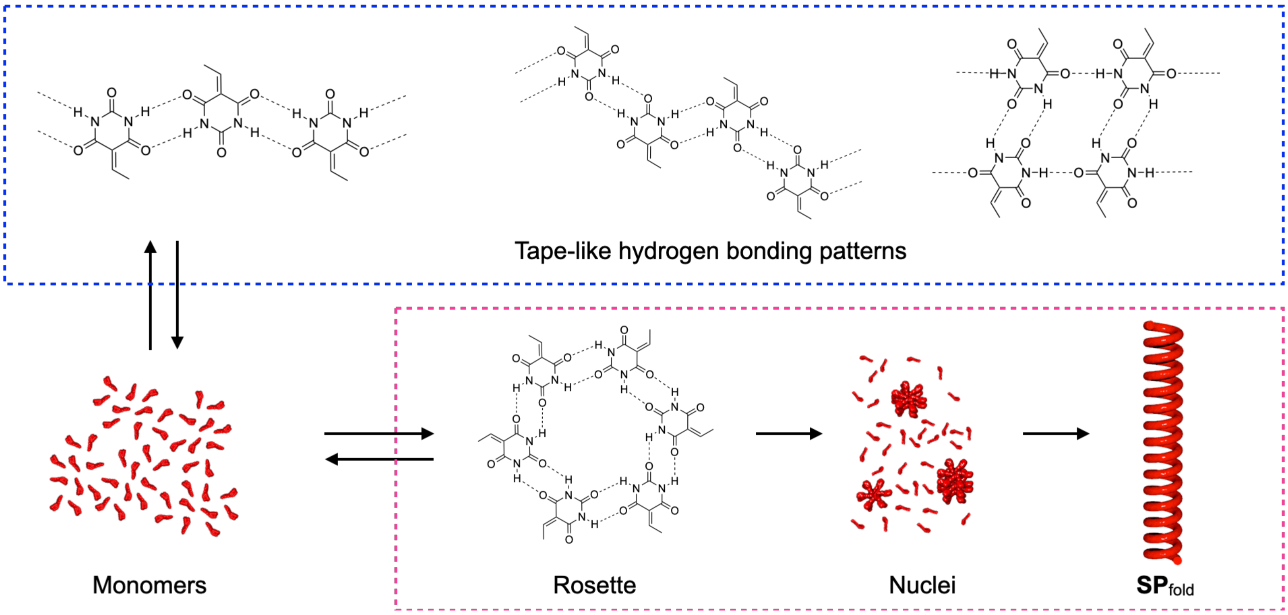

Figure S5. Competing hydrogen bonded assemblies upon supramolecular polymerization of barbiturate monomers. The formation of various hydrogen bonded assemblies is one of the reasons of the observed thermal hysteresis between cooling and heating curves.

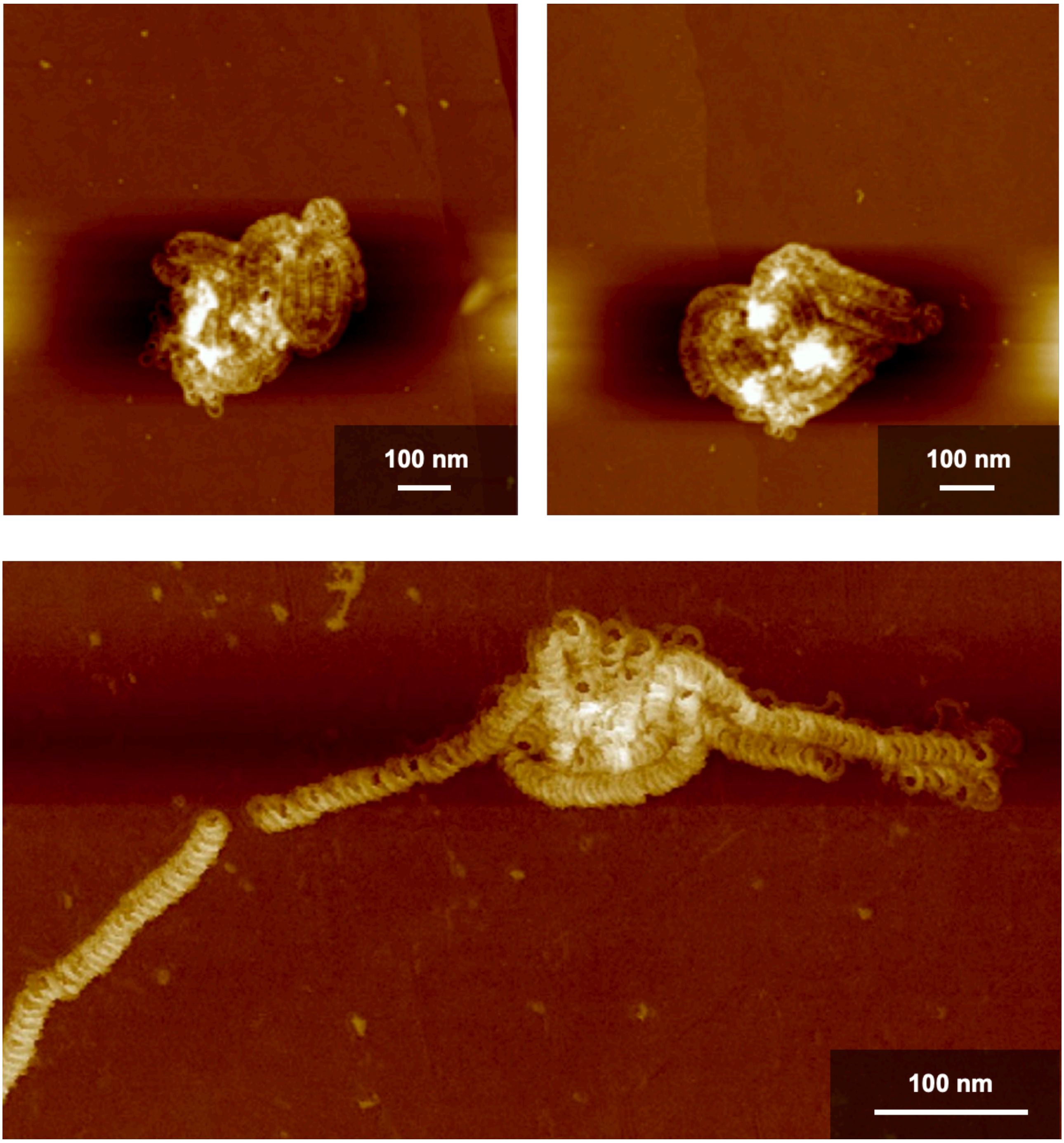

Figure S6. AFM images of $\mathbf{SP}_{\text{fold}}$ of **2** obtained upon cooling a monomer solution (c = 10 μM) from 373 to 308 K at the rate of 1.0 K min^{-1}.

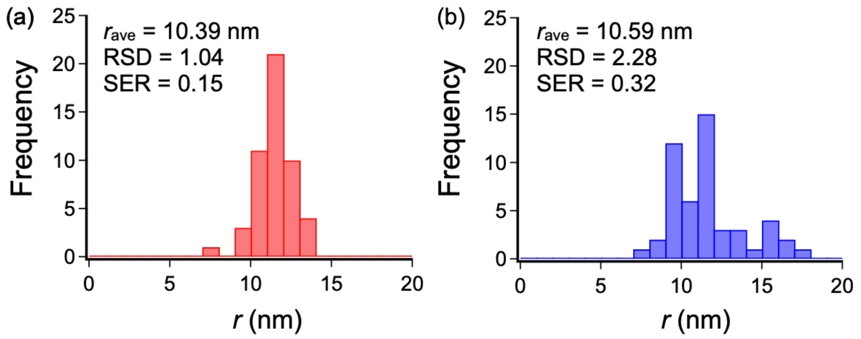

Figure S7. Distribution of curvature radii (r) estimated from AFM images of as-prepared **SP**$_{fold}$ (c = 10 μM) (a) and photo-unfolded **SP**$_{unfo}$ (b) of **2**, respectively. Insets: relative standard deviations (RSD) and standard errors (SER).

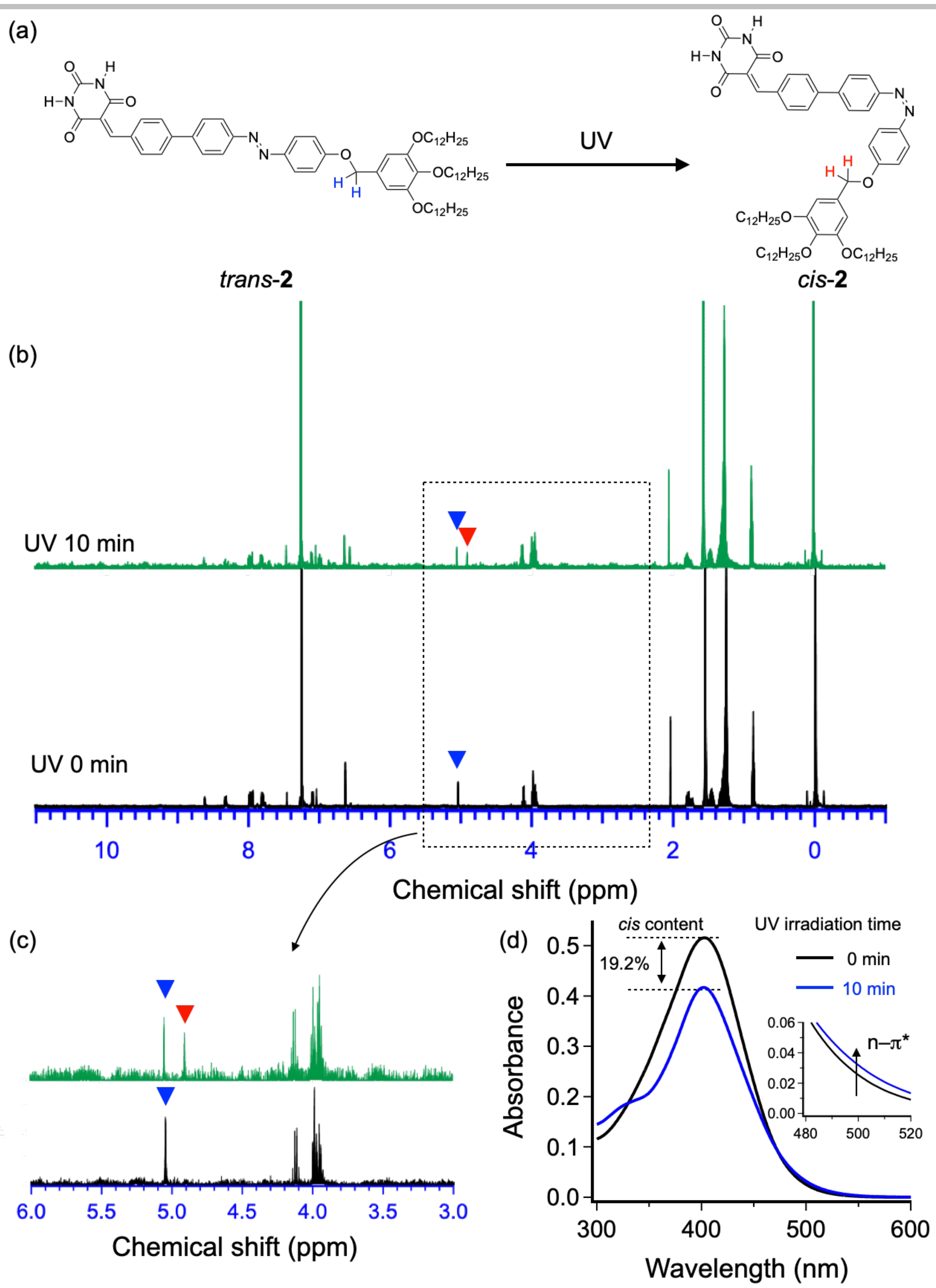

Figure S8. (a) Chemical structures of *trans*- and *cis*-isomers of **2**. (b) ^{1}H NMR spectra of monomeric *trans*-isomer of **2** (c = 1 mM) in $CDCl_3$ before and after UV-light irradiation for 10 min. The blue and red marks show signals of benzylic protons in *trans*- and *cis*-isomers, respectively. (c) Enlarged ^{1}H NMR spectra of the benzylic protons signals. (d) UV/vis absorption spectra of monomeric *trans*-isomer of **2** (c = 10 μM) in $CDCl_3$ before and after UV-light irradiation for 10 min. Inset: magnified view of absorption spectra around 500 nm wherein the n-π* transition of azobenzene is observed.

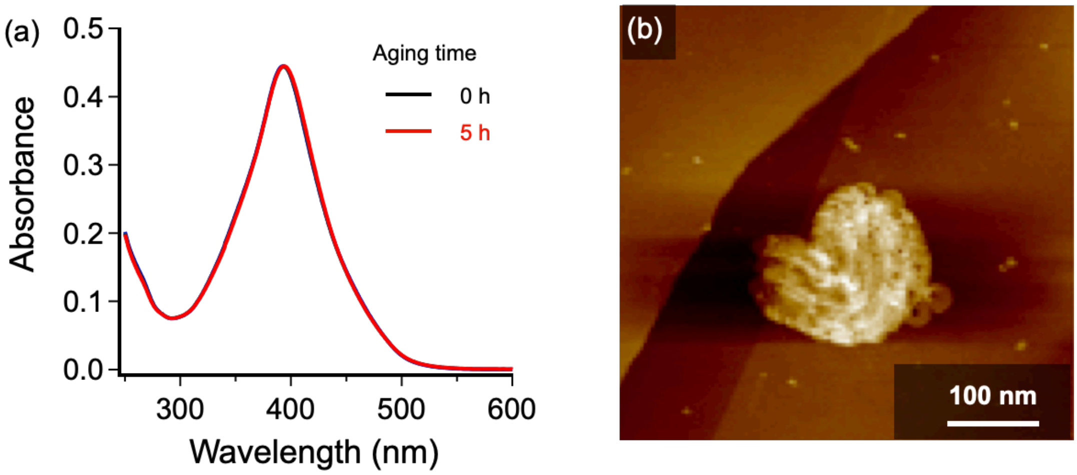

Figure S9. (a) UV/vis spectra and (b) AFM image of **SP**$_{\text{fold}}$ of **2** in MCH (c = 10 μM) after heating to 323 K and then aging for 5 h.

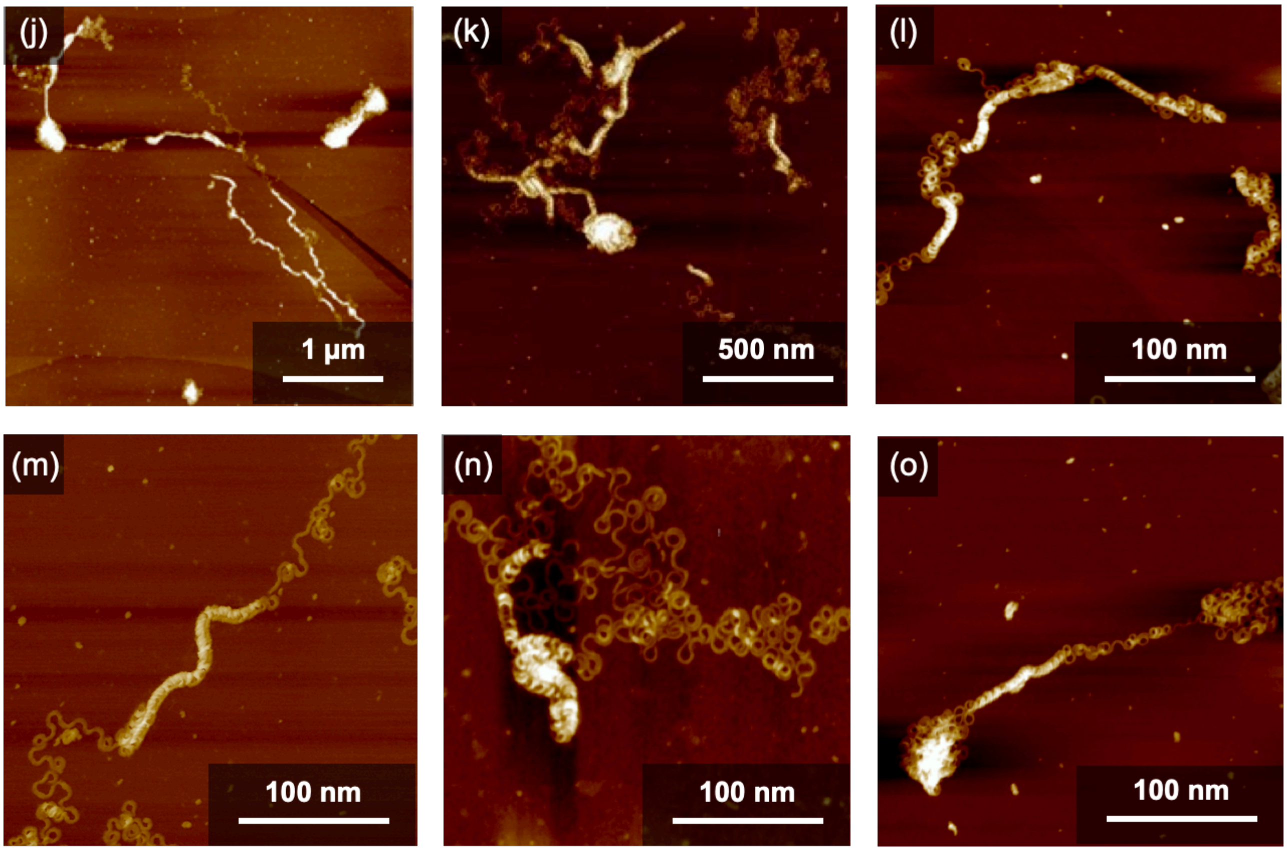

Figure S10. The original AFM images of Figure 3j–o in the main text.

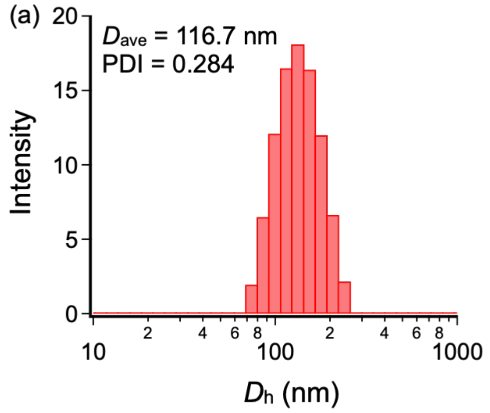

Figure S11. (a,b) Distribution of hydrodynamic diameters (D_h) of as-prepared $\mathbf{SP}_{fold}$ (c = 10 μM) (a) and photo-unfolded $\mathbf{SP}_{unfo}$ (b) of **2**, respectively. Insets: the average hydrodynamic diameter (D_H) values and the polydispersity index (PDI).

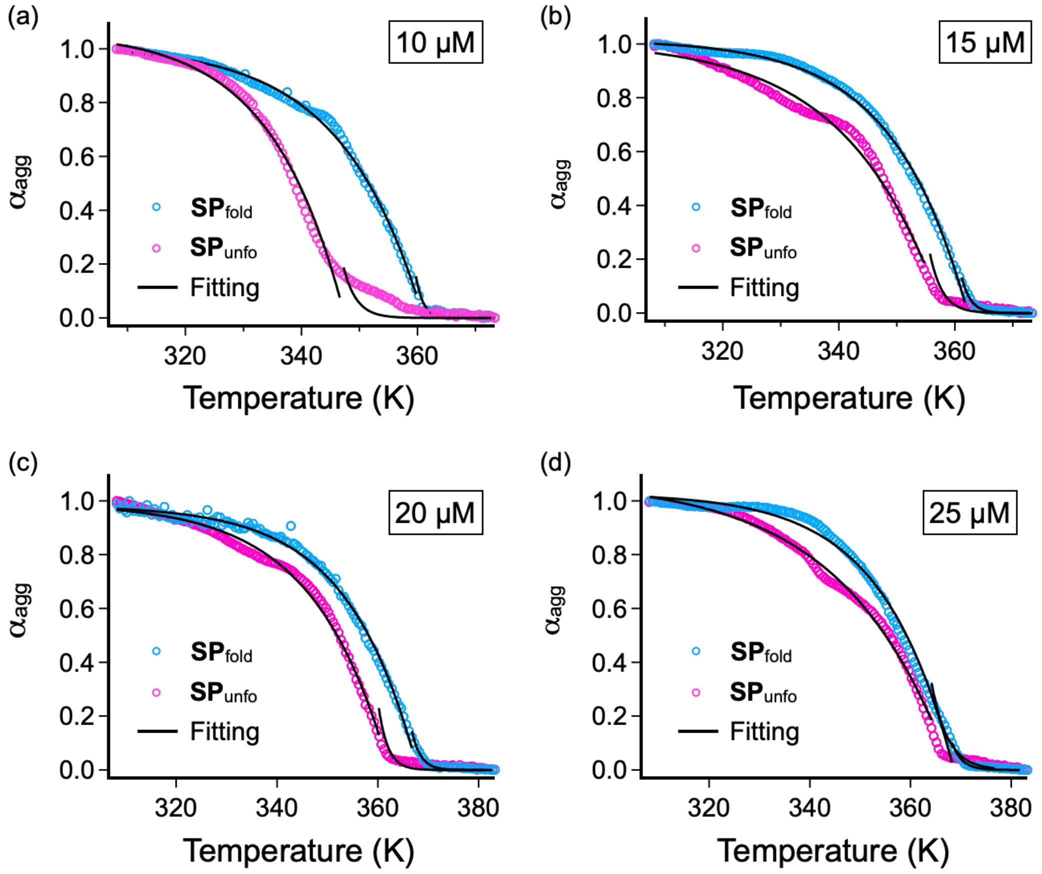

Figure S12. (a–d) Heating curves of $\mathbf{SP}_{fold}$ (blue circles) and unfolded $\mathbf{SP}_{unfo}$ (pink circles) of **2** in MCH at total concentration (c_T) of 10 (a), 15 (b), 20 (c), and 25 μM (d). Black solid curves were obtained by fitting of each curve with Supplementary Equations S2 and S3.

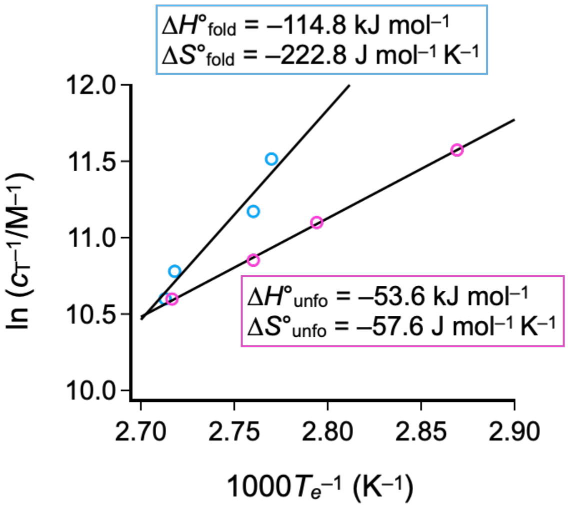

Figure S13. Modified van't Hoff plots of $\mathbf{SP}_{\text{fold}}$ (blue circles) and photo-unfolded $\mathbf{SP}_{\text{unfo}}$ (pink circles) of **2** using T_e obtained by the fitting analysis in Figure S12.

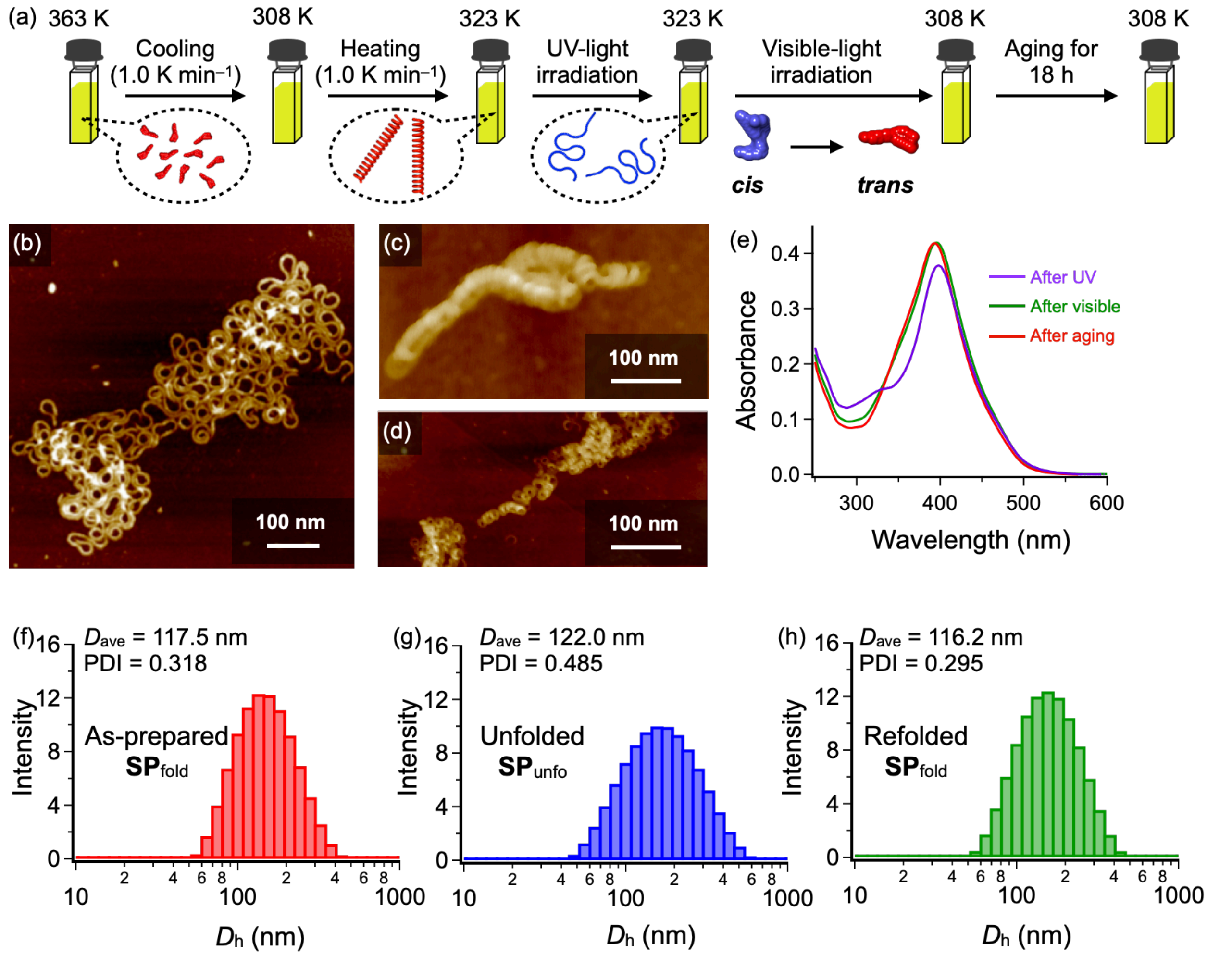

Figure S14. (a) Schematic representation of the procedure for a refolding experiment of **2** in MCH (c = 10 μM) by aging. (b–d) AFM images of SPs of **2** obtained just after visible-light irradiation for 10 min (b) and after subsequent aging at 308 K for 18 h (c,d). (e) UV/vis spectra of a MCH solution of SPs of **2** (c = 10 μM) after UV-light irradiation, subsequent visible-light irradiation, and further subsequent aging at 303 K for 18 h. (f–h) DLS size distributions of as-prepared **SP**$_{\text{fold}}$ (f), unfolded **SP**$_{\text{unfo}}$ by UV-light irradiation for 60 min at 323 K (g), and refolded **SP**$_{\text{fold}}$ of **2** (c = 10 μM) by subsequent visible-light irradiation for 10 min at 323 K and then aging for 18 h at 308 K (h). Insets: the average hydrodynamic diameter (D_H) values and the polydispersity index (PDI).

References

[1] T. F. A. De Greef, M. M. J. Smulders, M. Wolffs, A. P. H. J. Schenning, R. P. Sijbesma, E. W. Meijer, *Chem. Rev.* **2009**, *109*, 5687–5754.
[2] M. Wegener, M. J. Hansen, A. J. M. Driessen, W. Szymanski, B. L. Feringa, *J. Am. Chem. Soc*. **2017**, 139, 17979–17986.
[3] K. Aratsu, R. Takeya, B. R. Pauw, M. J. Hollamby, Y. Kitamoto, N. Shimizu, H. Takagi, R. Haruki, S. Adachi, S. Yagai, *Nat. Commun*. **2020**, *11*, Article number: 1623.
[4] B. Adhikari, Y. Yamada, M. Yamauchi, K. Wakita, X. Lin, K. Aratsu, T. Ohba, T. Karatsu, M. J. Hollamby, N. Shimizu, H. Takagi, R. Haruki, S. Adachi, S. Yagai. *Nat. Commun*. **2017**, *8*, Article number: 15254.
[5] J. Wang, R. M. Wolf, J. W. Caldwell, P. A. Kollman, D. A. Case, *J. Comput. Chem.* **2004**, *25*, 1157–1174.
[6] a) A. Jakalian, B. L. Bush, D. B. Jack, and C. I. Bayly, *J. Comput. Chem*. **2000**, *21*, 132–146; b) A. Jakalian, D. B. Jack, and C. I. Bayly, *J. Comput. Chem.* **2002**, *23*, 1623–1641.
[7] D. A. Case, H. M. Aktulga, K. Belfon, I. Y. Ben-Shalom, S. R. Brozell, D. S. Cerutti, T. E. Cheatham III, V. W. D. Cruzeiro, T. A. Darden, R. E. Duke, G. Giambasu, M. K. Gilson, H. Gohlke, A. W. Goetz, R. Harris, S. Izadi, S. A. Izmailov, C. Jin, K. Kasavajhala, M. C. Kaymak, E. King, A. Kovalenko, T. Kurtzman, T. S. Lee, S. LeGrand, P. Li, C. Lin, J. Liu, T. Luchko, R. Luo, M. Machado, V. Man, M. Manathunga, K. M. Merz, Y. Miao, O. Mikhailovskii, G. Monard, H. Nguyen, K. A. O'Hearn, A. Onufriev, F. Pan, S. Pantano, R. Qi, A. Rahnamoun, D. R. Roe, A. Roitberg, C. Sagui, S. Schott-Verdugo, J. Shen, C. L. Simmerling, N. R. Skrynnikov, J. Smith, J. Swails, R. C. Walker, J. Wang, H. Wei, R. M. Wolf, X. Wu, Y. Xue, D. M. York, S. Zhao, P. A. Kollman, Amber 2021, University of California, San Francisco, **2021**.
[8] L. Pesce, G. M. Pavan (2021, July 1). Research data supporting “Research data supporting: K. Tashiro, K. Katayama, K. Tamaki, L. Pesce, N. Shimizu, H. Takagi, R. Haruki, R. Heenan, M. J. Hollamby, G. M. Pavan, S. Yagai, "Topological Block Supramolecular Polymers Formed by Post-Supramolecular Polymerization Photo-Unfolding", under submission". Zenodo. http://doi.org/10.5281/zenodo.5025104
[9] B. Hess, C. Kutzner, D. van der Spoel, E. Lindahl, *J. Chem. Theory Comput.* **2008**, *4*, 435–447.
[10] G. A. Tribello, M. Bonomi, D. Branduardi, C. Camilloni, G. Bussi, *Comput. Phys. Commun.* **2014**, *185*, 604–613.
[11] G. Bussi, D. Donadio, M. Parrinello, *J. Chem. Phys*. **2007**, *126*, 014101.
[12] H. J. C. Berendsen, J. P. M. Postma, W. F. van Gunsteren, A. DiNola, J. R. Haak, *J. Chem. Phys.***1984**, *81*, 3684–3690.
[13] U. Essmann, L. Perera, M. L. Berkowitz, T. Darden, H. Lee, L. G. Pedersen, *J. Chem. Phys*. **1995**, *103*, 8577–8593
[14] B. Hess, H. Bekker, H. Berendsen, J. Fraaije, *J. Comput. Chem.* **1997**, 18, 1463–1472.

Author Contributions

Keigo Tashiro (writing of original draft, data curation, formal analysis, investigation), Kosuke Katayama (data curation, formal analysis, investigation), Kenta Tamaki (writing of original draft), Luca Pesce (writing of original draft, data curation, formal analysis, investigation), Nobutaka Shimizu (data curation, formal analysis, investigation), Hideaki Takagi (data curation, formal analysis, investigation), Rie Haruki (data curation, formal analysis, investigation), Martin J. Hollamby (writing of original draft, formal analysis, investigation) Giovanni M. Pavan (writing of original draft, data curation, formal analysis, investigation), Shiki Yagai (writing of original draft, project administration, funding acquisition)
